# Stress‐sensitive dynamics of miRNAs and Elba1 in *Drosophila* embryogenesis

**DOI:** 10.15252/msb.202211148

**Published:** 2023-03-20

**Authors:** Lovisa Örkenby, Signe Skog, Helen Ekman, Alessandro Gozzo, Unn Kugelberg, Rashmi Ramesh, Srivathsa Magadi, Gianluca Zambanini, Anna Nordin, Claudio Cantú, Daniel Nätt, Anita Öst

**Affiliations:** ^1^ Department of Biomedical and Clinical Sciences Linköping University Linköping Sweden; ^2^ Division of Neurobiology (NEURO) Linköping University Linköping Sweden; ^3^ Division of Molecular Medicine and Virology (MMV) Linköping University Linköping Sweden

**Keywords:** *Drosophila*, Elba1, Embryogenesis, miRNA/MZT, Chromatin, Transcription & Genomics, Development

## Abstract

Early‐life stress can result in life‐long effects that impact adult health and disease risk, but little is known about how such programming is established and maintained. Here, we show that such epigenetic memories can be initiated in the *Drosophila* embryo before the major wave of zygotic transcription, and higher‐order chromatin structures are established. An early short heat shock results in elevated levels of maternal miRNA and reduced levels of a subgroup of zygotic genes in stage 5 embryos. Using a *Dicer‐1* mutant, we show that the stress‐induced decrease in one of these genes, the insulator‐binding factor Elba1, is dependent on functional miRNA biogenesis. Reduction in Elba1 correlates with the upregulation of early developmental genes and promotes a sustained weakening of heterochromatin in the adult fly as indicated by an increased expression of the PEV *w* ^*m4h*^ reporter. We propose that maternal miRNAs, retained in response to an early embryonic heat shock, shape the subsequent *de novo* heterochromatin establishment that occurs during early development via direct or indirect regulation of some of the earliest expressed genes, including Elba1.

## Graphical abstract

Heat shock during *Drosophila* embryogenesis results in the retention of maternal miRNAs and the downregulation of pre‐MBT genes, including Elba1, which may contribute to setting the stage for subsequent gene expression.

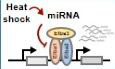

## Introduction

Early life is carefully orchestrated by a plethora of processes that allow for both developmental robustness and plasticity, ultimately regulating the diversity of phenotypes from a single genome. This provides the foundation for the Developmental Origins of Health and Disease (DOHaD) hypothesis, which postulates that the etiologies of major public health issues, such as obesity, type 2 diabetes, and heart disease, depend on suboptimal conditions during sensitive periods early in life (Suzuki, 2018). In humans, this can be caused by factors such as malnutrition, smoking, physical, or psychological trauma (reviewed in Knopik *et al*, 2012; Cunliffe, 2016; Wong & Langley, 2016; Block & El‐Osta, 2017), in *Arabidopsis* by hyperosmotic stress (Sani *et al*, 2013) and in *Drosophila melanogaster* by, e.g., heat shock (Seong *et al*, 2011). The developmental timing of exposure has proven crucial for determining the outcome and, to date, it is poorly understood what sets such sensitive developmental periods apart from insensitive ones. Moreover, the molecular mechanisms initiating and shaping the response, as well as how memories of these exposures are kept throughout the developmental reorganization of the chromatin landscape, remains to be understood.

*Drosophila* embryogenesis is extremely rapid with < 3 h from fertilization to gastrulation and only 22–27 h to the first larvae stage. The main reason for this is that the early *Drosophila* embryo, like most insects, undergoes a series of rapid mitotic events without cytokinesis where all nuclei share the same cytoplasm (reviewed in Hamm & Harrison, 2018). These cycles, which are only separated by a few minutes, are too short for extensive zygotic transcription (De Renzis *et al*, 2007; Kwasnieski *et al*, 2019) making these precellular stages of *Drosophila* embryogenesis highly dependent on maternally loaded proteins and RNAs. At the midblastula transition (MBT), there is a lengthening and synchronization of mitotic cycles that coincide with the zygotes' claim of transcriptional independence, a process crucial for the maternal‐to‐zygotic transition (MZT; Vastenhouw *et al*, 2019). Before MZT, there are no higher‐order chromatin organization reported. During MZT, however, several well‐coordinated events, driven by an interplay between maternally provided products and zygotic *de novo* transcription, lead to the establishment of chromatin states and a chromosomal 3D architecture that can be detected by Hi‐C as topologically associated domains (TADs; Li *et al*, 2014; Yuan *et al*, 2016; Hug *et al*, 2017; Stadler *et al*, 2017; Hamm & Harrison, 2018). One important component for establishing a higher‐order chromatin structure is insulator‐binding factors that bind to genomic cis‐regulatory insulator sequences to prevent leakage of the regulatory environment between neighboring genes and across longer distances (Stadler *et al*, 2017). Recently, a family of insulator‐binding proteins was discovered, the Elba complex, expressed just before the MBT to ensure the partition of transcription units during the transition to zygotic independence (Aoki *et al*, 2012; Ueberschär *et al*, 2019).

That transcription of early zygotic microRNA (miRNA) is important for the degradation of maternal transcripts has been known for more than a decade (Bushati *et al*, 2008). In addition, miRNA together with other small noncoding RNAs (sncRNAs), including piwi‐interacting RNA (piRNA), fragments of tRNA (tsRNA), and rRNA (rsRNA), has been shown to play important roles in inter‐ and transgenerational epigenetic inheritance (de Castro Barbosa *et al*, 2015; Grandjean *et al*, 2015; Sharma *et al*, 2016; Zhang *et al*, 2018; Nätt *et al*, 2019). In combination with the known involvement of siRNA in heterochromatin formation (Li *et al*, 2009b), piRNA in transposon silencing (Huang *et al*, 2013), and certain tRNA halves in regulating histone biogenesis (Boskovic *et al*, 2019), it is easy to envision a general role for sncRNA, in initiating or influencing the early higher‐order chromatin landscape (Holoch & Moazed, 2015; Johnson & Straight, 2017; Allshire & Madhani, 2018). Furthermore, cellular responses to stress involve the upregulation and activation of specific miRNA (Leung & Sharp, 2010; Olejniczak *et al*, 2018) and proteins (Chen *et al*, 2018), as well as fragmentation of tRNA (Thompson *et al*, 2008). Thus, in addition to a central role in initiating chromatin states, sncRNA plays a vital role in the cellular stress response. Currently, there are no fine‐resolution data of sncRNA covering the first stages of embryogenesis.

Here, we explore the effects of environmental stress on the expression of sncRNA during early *Drosophila* embryogenesis. We specifically aimed to identify sensitive developmental windows in which stress might induce long‐lasting memories. Furthermore, by examining gene‐ and sncRNA expression within the same single *Drosophila* embryos, we aimed to identify critical interactions between sncRNA and genes in such a sensitive window.

As previously shown (Hartmann‐Goldstein, 1967; Lu *et al*, 1998; Seong *et al*, 2011; Bughio *et al*, 2019), we find that heat shock before the MBT reduces the epigenetic‐mediated, H3K9/H3K20 methylation‐dependent silencing of the position‐effect variegation (PEV) sensor *w* ^*m4h*^, which is an adult eye color heterochromatin reporter (Elgin & Reuter, 2013). Such early heat shock results in the retention of maternally loaded miRNAs in the embryo, including a specific group of miRNA that negatively associates with the expression of some of the earliest transcribed genes. Finally, frame‐shift mutation of one of these genes, *Elba1* (a.k.a. *Bsg25A*) and its partners Elba 2 and 3, efficiently mimicked the effect of heat shock on the adult eye color reporter, thus suggesting that a temporal expression of embryonic insulators have a long‐lasting epigenetic effect.

## Results

### Heat shock during the first 2 h of embryogenesis causes long‐term effects

**Figure 1:**
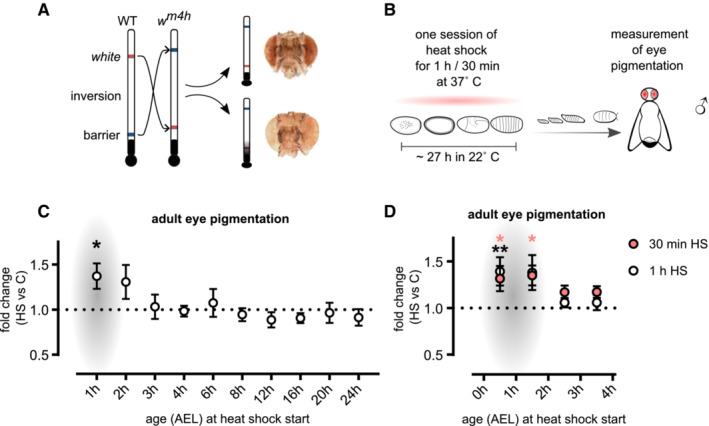
The most sensitive period for heat shock‐induced epigenetic programming is the first 2 h of embryogenesis A
*Drosophila w* ^*m4h*^ has an inversion of the *white* gene, needed for eye pigmentation, which places this gene in proximity to the centromeric heterochromatin. This enables the detection of heterochromatin spreading through the measurement of eye pigmentation.B
Eggs were collected at 1 h intervals and exposed to one session of 1 or 0.5 h heat shock at different developmental time windows or kept as controls. Developing flies were kept at 22°C until pupae hatching, and eye pigmentation was measured in 5‐day‐old males.C, D
Eye pigmentation in relation to controls (not exposed to heat shock). The most stress‐sensitive period was found during the first hour of embryogenesis. At this time point, a 1 h (C, D) or 30 min (D) heat shock resulted in adult flies with more pigmented eyes, indicative of more open chromatin at this locus. Heads were measured in groups of 3–10 and normalized to the average optical density per head. Data information: Number of pools per time window (same order as in graph) = 9, 9, 12, 12, 14, 10, 8, 11, 8, 7 (C) and *n* = 17, 14, 18, 17 for 30 min heat shock (D) and *n* = 13, 7, 10, 8 for 1 h heat shock (D). AEL, after egg laying. Presented with ± SEM, * (adjusted *P* = 0.0329 (C), 0.0259 and 0.0188 (D)) and ** (adjusted *P* = 0.0075) with ordinary one‐way ANOVA with the Dunnett's multiple comparison test. Red asterisk = 30 min heat shock, black asterisk = 1 h heat shock.

To identify sensitive periods during *Drosophila* development, where stress exposure can induce long‐term memory, we used the position‐effect variegation strain *w* ^*m4h*^ (Fig 1A). This strain has an inversion on the X chromosome, positioning the *white* gene close to the pericentric heterochromatin. The expression of *white*, which is needed for eye pigmentation, is therefore controlled by the centromeric chromatin state and the adult eye color can be used as a reporter for heterochromatin at this locus (Elgin & Reuter, 2013). Variegation of *w* ^*m4h*^ is controlled by the methyltransferases Su(var) 3–9, Su(var) 4–20, E(z), and HP1 (Phalke *et al*, 2009). In addition, we have previously showed that this reporter is sensitive to paternal diets (Öst *et al*, 2014).

To enable high‐resolution mapping of sensitive periods for environmentally induced changes in variegation, we used a heat shock intervention. In contrast to other environmental challenges such as suboptimal nutrition and exposure to toxins, heat shock allows for a sharp and distinct intervention time. We performed a 1‐h heat shock during different time points of *Drosophila* development, throughout embryogenesis (Fig 1B and C), as well as during the larva stages (Appendix Fig S1). After assessing the eye color in 5‐day‐old male adults, we found that the first 2 h in embryogenesis is the only sensitive period for heat shock induction of long‐term effects on heterochromatin (Fig 1C; Appendix Fig S1). This finding was confirmed by repeating the experiment with an even shorter heat shock exposure (30 min; Fig 1D). Our finding is consistent with previous work identifying the first 0–3 h after fertilization as a time in which the epigenome is sensitive to heat shock stress (Hartmann‐Goldstein, 1967; Lu *et al*, 1998; Seong *et al*, 2011; Bughio *et al*, 2019). Importantly, we performed heat shock in more narrow intervals than previously reported (Seong *et al*, 2011), and while we did notice effects in 0–1 and 1–2 h, we did not detect any significant long‐lasting effects on *white* expression in 2‐ to 3‐h‐old embryos, indicating that in order for long‐term effects to occur, the exposure of a stressor must happen before the MBT.

### Early *Drosophila* embryogenesis is accompanied by dynamic shifts in sncRNA

**Figure 2:**
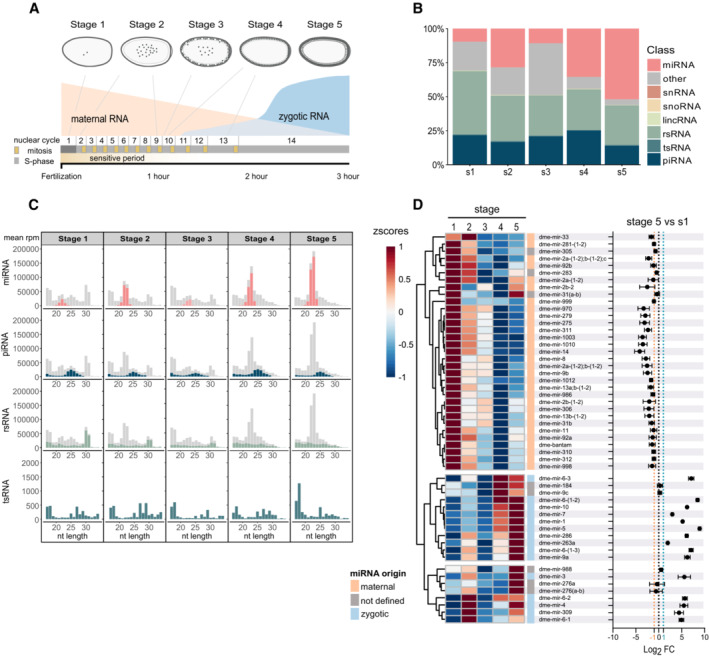
Early embryogenesis is characterized by rapid changes in sncRNA Schematic illustration of the first 5 stages of *Drosophila* embryogenesis.Relative proportions of sncRNA classes obtained after the sequencing of *w* ^*m4h*^ embryos of stages 1–5. *n* = 5 (stages 1–3), *n* = 4 (stages 4–5) embryos. rsRNA = rRNA fragments, tsRNA = tRNA fragments.Mean rpm per nucleotide length and stage. Color represents the indicated sncRNA class and gray all other sncRNA. *n* = 5 (stages 1–3), *n* = 4 (stages 4–5) embryos.(Left) miRNA expression (zscores of mean rpm) per stage. The clusters based on expression profiles separate maternal and zygotic miRNA. (Right) Log2 fold change of rpm per indicated miRNA between mean stage 5 and stage 1 embryos. Fold change ≤ −1—maternal, ≥ 1—zygotic, and between—not defined origin. Classifications were made in accordance with the unique miRNA sequence expression profiles and available spike‐in normalized miRNA expression data (Zhou *et al*, 2018). Results are mean ± SD. *n* = 5 (stages 1–3), *n* = 4 (stages 4–5) embryos.

The first 2 h of *Drosophila* embryogenesis, entailing stages 1–3 (at 22°C), is characterized by rapid mitotic cycles dependent on maternally loaded mRNAs and proteins (Fig 2A; Bushati *et al*, 2008; Tadros & Lipshitz, 2009; Vastenhouw *et al*, 2019). In concordance with the rapid cell divisions, there is no clear higher‐order chromatin architecture in this period (Li *et al*, 2014; Hug *et al*, 2017; Ogiyama *et al*, 2018). Chromatin states are fully established at MBT, around stage 5, when the tempo of mitosis subsides and zygotic transcription is activated (Rudolph *et al*, 2007; Zenk *et al*, 2021). As sncRNAs are highly present already in stages 1–3 and are known to modulate higher‐order chromatin structure, we hypothesized that heat shock‐induced changes of sncRNA might precede and guide the *de novo* heterochromatin formation. As there are no high‐resolution timelines of changes in sncRNA during these early stages of *Drosophila* embryogenesis, we began by performing sncRNA sequencing on single embryos, embracing stages 1–5 (Fig 2A). We found that the relative proportions (Fig 2B) and size distributions (Fig 2C) of different sncRNA classes were highly dynamic during these stages.

As expected during the maternal‐to‐zygotic transition, stage 3 is characterized by an increased proportion of degradation products (“other,” Fig 2B), which then decrease at the end of MZT. We also noted that there is a larger proportion of rRNA fragments (rsRNAs) in stage 1 than in the other stages, while tRNA fragments (tsRNAs) are present at low levels at all stages (Fig 2B and C). By contrast, piRNAs are highly expressed throughout all stages. The most striking change, however, was the proportion of miRNA. It increased from approximately 9% in stage 1 embryos to over 50% in stage 5 (Fig 2B).

While multiple miRNAs were reduced during this developmental time window, suggestive of a maternal origin, the zygotic mir‐309 cluster and a few other miRNAs showed a pronounced upregulation (Fig 2D). Controlled by Zelda, a maternally provided pioneering transcription factor (Liang *et al*, 2008; Fu *et al*, 2014), the mir‐309 cluster plays an important role in the zygotic‐driven pathway that degrades maternal transcripts (Bushati *et al*, 2008). Sequential comparison of stage 2 against 1, 3 against 2, 4 against 3, and 5 against 4 revealed that even though many transcripts from the mir‐309 cluster had a sharp increase in stages 4 and 5 (Appendix Fig S2C and D), there were several members of this cluster that showed a significant increase already between stage 1 and 2 (Fig 2D; Appendix Fig S2A). To test that this early activation was not an artifact driven by a few outlier miRNA sequences, we compared unique miRNA sequences per sample and stage (Appendix Figs S2 and S3; Dataset EV1). This revealed an upregulation of several unique miRNA sequences of the mir‐309 cluster and strengthened the notion that there is an upregulation of this cluster between stages 1 and 2. Previous findings have shown that members of this cluster are expressed at low levels in 0‐ to 1‐h‐old embryos and are strongly induced 2–3 h after egg laying (Aravin *et al*, 2003; Ruby *et al*, 2007; Bushati *et al*, 2008; Fu *et al*, 2014; Ninova *et al*, 2014). Furthermore, low levels of Zelda have been detected in the nucleus already at nuclear cycle 2 (Nien *et al*, 2011). Our results align with these findings and support a scenario where some members of the mir‐309 cluster are starting to be transcribed at low levels already between embryonic stages 1 and 2.

### Heat shock in the sensitive period results in a rapid change of sncRNA at the time of *de novo* heterochromatin formation

**Figure 3:**
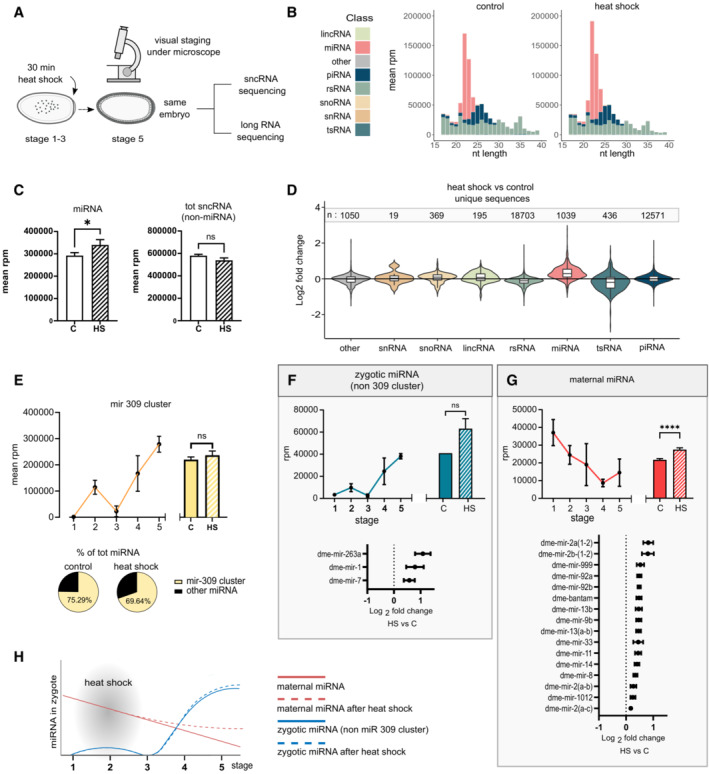
Stress‐induced upregulation of miRNA A
*Drosophila w* ^*m4h*^ embryos were collected in short intervals (30 min) and immediately heat‐shocked for 30 min at 37°C (or kept as controls). After being manually staged under a microscope, RNA from the same embryos was used for sequencing of both sncRNA and rRNA‐depleted long RNA.B
Read‐length distributions of sncRNA from heat‐shocked and control *w* ^*m4h*^ embryos of all sncRNA classes obtained after the sequencing of sncRNA. *n* = 24 embryos per condition.C
Expression of total miRNA (left), and all other sncRNA (right) between conditions. Results are mean ± SEM of 24 samples per condition, *P = 0.0389 using an unpaired one‐tailed Mann–Whitney test.D
Log_2_ fold change of unique reads per sncRNA class between heat‐shocked and control samples. *n* = number of unique sequences per sncRNA class. Log2 fold change is based on 24 embryos per condition.E
Total expression of the mir‐309 cluster per stage (left), *n* = 5 (stages 1–3), = 4 (stages 4–5), or per condition (right), *n* = 24 single embryos. Bar graphs are mean ± SEM, ^ns^ *P* = 0.1305 using an unpaired two‐tailed Mann–Whitney test.F, G
Total expression of the zygotic (non‐mir‐309) (F) or maternal (G) miRNA per stage (top left), *n* = 4 (stages 1–3), = 5 (stages 4–5), or per condition (top right), *n* = 24 single embryos. Bar graphs are mean ± SEM, ****P < 0.0001 using an unpaired one‐tailed Mann–Whitney test, ^ns^ *P* = 0.0508. (bottom) Log_2_ fold changes of significantly changed FDR‐corrected ^ns^ *P* = 0.0508 < 0.05 using the DEseq2's build‐in Wald test after negative binominal fitting of indicated miRNA between heat‐shocked and control embryos. Results are mean ± SEM.H
Schematic illustration of maternal and zygotic miRNA levels during the first 5 stages of embryogenesis with or without heat shock exposure. The illustration is based on our observations from miRNA expression profiles during stages 1–5 (Fig 2D) and (F, G).

**Figure EV1:**
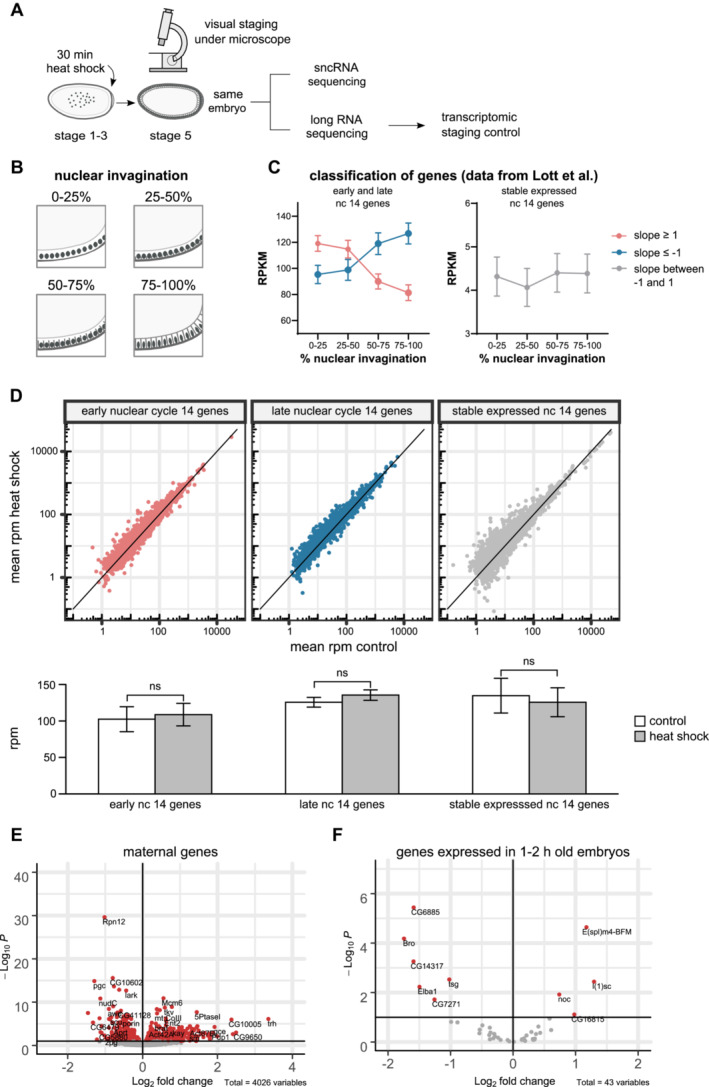
A heat shock does not induce a temporal bias in the embryonic transcriptome A
*Drosophila w* ^*m4h*^ embryos were collected in short intervals (30 min) and immediately heat‐shocked for 30 min at 37°C (or kept as controls). After being manually staged under a microscope, RNA from the same embryos was used for sequencing of both sncRNA and rRNA‐depleted long RNA.B, C
Transcriptome from 4 chronological parts of the nuclear cycle (nc) 14 by Lott *et al* (2011) were subjected to linear regression. (C) Genes were defined as expressed early (slope ≥ 1), late (slope ≤ −1), or stable (slope between −1 and 1) Results are mean ± SEM RPKM.D
(top) Transcriptome of heat‐shocked and control embryos from (A) were separated into early, late, and stable genes defined in (C). (bottom) Bar graph of mean rpm of early, late, or stable genes from control and heat‐shocked embryos. The nonsignificant (ns) result is based on the unpaired, two‐tailed *t*‐test. Results are mean ± SD of *n* = 24 embryos per condition.E
Maternally provided genes as classified in Lott *et al* (2011) showed an equal distribution of up‐ (*n* = 2,133), respectively, downregulated (*n* = 1,893) genes between heat‐shocked and control embryos. Results are Log2 fold change (heat shock vs. control) of *n* = 24 embryos per condition.F
Early zygotic genes (1‐2 h) as classified in De Renzis *et al* (2007) showed an equal distribution of up (*n* = 21), respectively, downregulated (*n* = 22) genes. Results are Log2 fold change (heat shock vs. control) of *n* = 24 per heat shock or control condition.

To investigate whether a heat shock in the identified sensitive period results in changes to the sncRNA‐profile at cellularization and *de novo* heterochromatin formation, we next heat‐shocked 0‐ to 0.5‐h‐old embryos for 30 min and then aged them to stage 5 (Figs 3A and EV1). Precisely hand‐staged single embryos—with completed cellularization typical for the later stage 5 (Bownes, 1975)—were selected for sequencing (Fig 3A). As the gene expression is very dynamic during embryonic cellularization and gene activation, we used two strategies to ensure that minor mistakes during staging would not influence the data. First, we ensured a good sample size by selecting 24 embryos for each condition. Second, in parallel to the sncRNA‐seq, we performed rRNA‐depleted sequencing of long RNA from the same single embryo. Since there is published gene expression data of stage 5 (nuclear cycle 14) that is chronologically divided into four parts (Lott *et al*, 2011), we reasoned that it could be used to control our staging. By looking at the linear regression of the four parts of stage 5 gene expression, we classified genes as early (slope ≤ −1), late (slope ≥ 1), or stably expressed (slope between −1 and 1; Fig EV1B and C). We could not detect any temporal bias between our groups using this classification (Fig EV1D). Neither could we detect any temporal bias between our groups when we compared it against the expression of maternally provided (Fig EV1E; Lott *et al*, 2011) nor early zygotic genes (transcribed within 1–2 h of embryogenesis; Fig EV1F; De Renzis *et al*, 2007). Thus, we concluded that there was no temporal bias between control and heat‐shocked embryos.

**Figure EV2:**
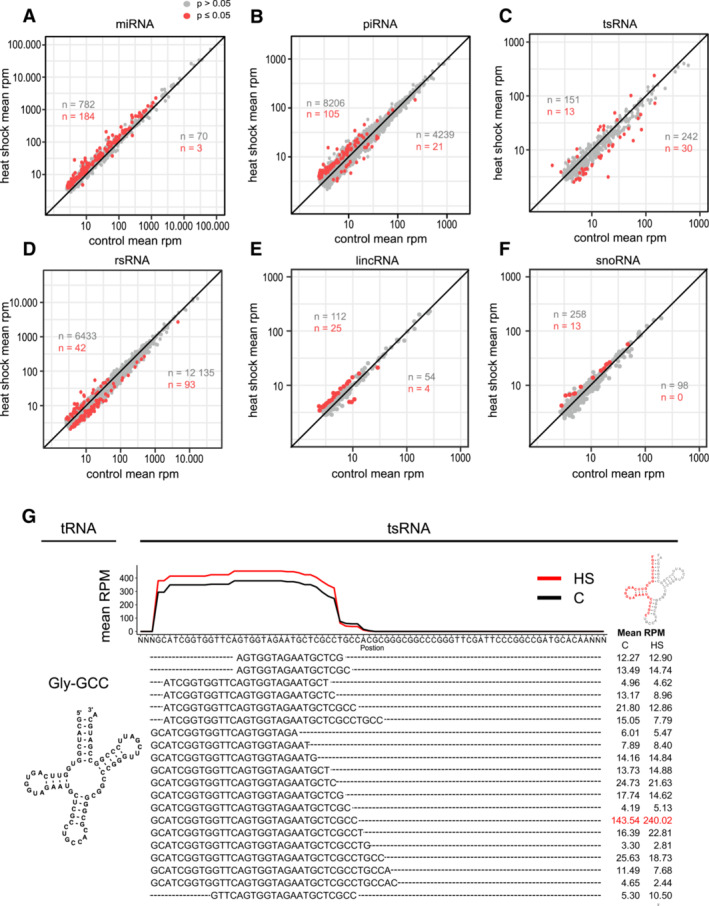
Unique sncRNA sequences after exposure to heat shock during the sensitive period A–F
Scatter plots comparing rpm normalized expression levels of unique miRNAs (A), piRNAs (B), tRNA‐ (C) rRNA fragments (D), lincRNAs (E), snoRNAs (F) between control and heat‐shocked embryos. Red = FDR corrected *P* ≤ 0.05, gray = FDR corrected *P* > 0.05 using the DEseq2's build‐in Wald test after negative binominal fitting.G
Line graph shows mean rpm coverage of reads mapping to tRNA‐Gly‐GCC. Mean rpm of each unique sequence contributing to the line plots is presented below the graph. The most significantly differentially expressed sequence is marked in red. Data information: FDR corrected *P* ≤ 0.05 using the DEseq2's build‐in Wald test after negative binominal fitting. C, control sample; HS, heat‐shocked. *n* = 24 single *w* ^*m4h*^ embryos per condition.

Analysis of the sncRNA‐seq data showed, as earlier (Fig 2B and C), that rRNA fragments and miRNAs (Fig 3B) dominate the sncRNA profiles of stage 5 embryos. We did not detect any changes in size distribution between the conditions (Fig 3B), but we found a significant increase of miRNAs in heat‐shocked samples (Fig 3C), a result of several upregulated unique miRNA sequences (Figs 3D and EV2A; Datasets EV2 and EV3). In addition, differential expression analysis of all unique reads revealed upregulation of several piRNAs, lincRNAs, and snoRNAs (Fig EV2B, E, and F), while tsRNA and rsRNA showed diverse responses (Fig EV2C and D). As expected, we found that heat shock induced more tRNA halves originating from the 5′ terminal of mature Gly‐GCC (Fig EV2G). This tsRNA, which is also called tRNA‐derived stress‐induced small RNA, is cleaved at the anticodon loop by ribonucleases like angiogenin and is generated in response to different kinds of cellular stress leading to the formation of stress granules (Emara *et al*, 2010). In mammals, such tRNA 5′ halves are known to be changed in sperm in response to diet and to modulate early embryonic processes (Chen *et al*, 2016; Nätt & Öst, 2020), but their role in *Drosophila* embryogenesis is unknown. Nonetheless, the most prominent effect of early embryonic heat shock on stage 5 embryos was the significant increase of 184 unique miRNA sequences, indicative of a miRNA‐dependent stress response preceding MBT and zygotic gene activation (ZGA). Importantly, we did not detect any difference in the expression of the miR‐309 cluster, accounting for 69–75% of total miRNA at stage 5 (Fig 3E).

The heat shock‐induced upregulation of miRNA could be due to either increased zygotic transcription or decreased degradation of maternal transcripts. These two scenarios were discriminated by comparing the miRNA profiles from stages 1 to 5 embryos (Figs 2D and 3F and G, Appendix Fig S2 and S3). Since we found a clear distinction between the miRNA species found at the first stage compared with stages 4–5 (Fig 2D; Appendix Fig S3), we classified miRNAs having their peak expression during stage 1 as maternally loaded and miRNAs that had their peak in later stages as zygotic. To confirm that this classification was correct, we compared our results with the miRNA expression profiles of 0‐ to 2‐ and 2‐ to 4‐h‐old embryos made by Zhou *et al* (2018) and found that our classifications largely align with theirs. Using this division, we found that only a few zygotic miRNAs increased in response to heat shock, whereas there was a distinct increase in maternally provided miRNAs (Fig 3F–H).

### The pre‐MBT insulating binding factor Elba1 is downregulated in response to heat shock in a *Dicer‐1*‐dependent manner

**Figure 4:**
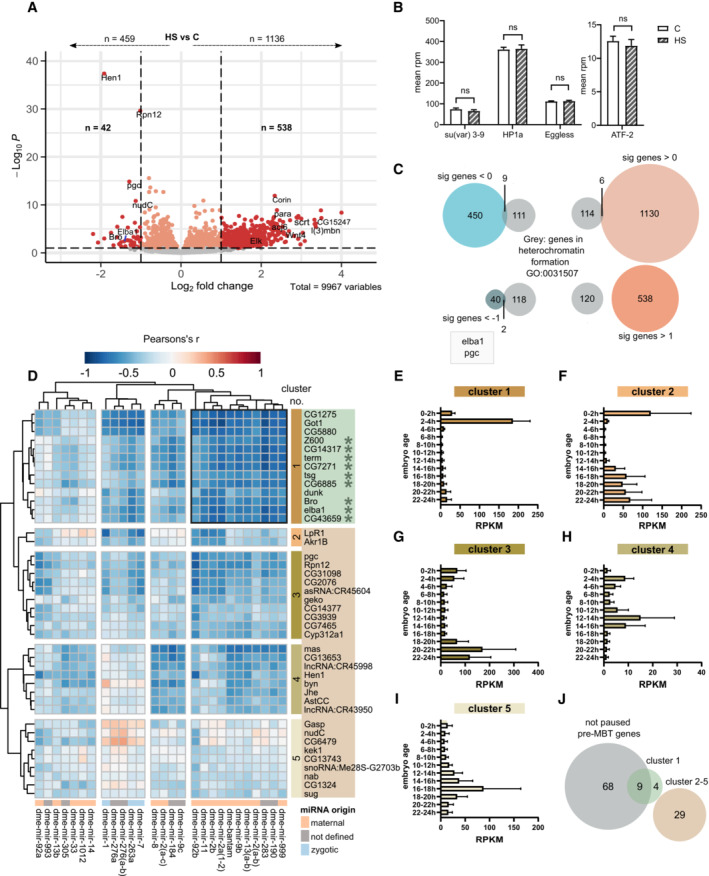
Heat shock reduces specific pre‐MBT genes A
Volcano plot showing differentially expressed long RNA in sequenced heat‐shocked compared with control *Drosophila w* ^*m4h*^ stage 5 embryos. Dark and light red indicates significance at *P* ≤ 0.05 (FDR corrected *P*‐values using the DEseq2's build‐in Wald test after negative binominal fitting) and dark red a log2 fold change ≥ or ≤ ±1.B
No significant changes of *Su(var) 3–9*, *Eggless*, *HP1a*, or *ATF‐2* were detected after early embryonic heat shock. Results are mean ± SEM, ns = nonsignificant using the multiple unpaired *t*‐test. *n* = 24 embryos per condition.C
Intersections between differentially expressed genes and genes from the *Drosophila* gene ontology term heterochromatin formation (GO:0031507). Two significantly downregulated (< −1) genes (elba1 and pgc) are highlighted.D
Pearson's *r* for all significantly upregulated miRNAs and downregulated long RNAs. Unsupervised Euclidean clustering shows that several maternal miRNAs correlate strongly negatively to gene cluster 1.E–I
Temporal expression of each gene cluster using modENCODEs data. Results are mean ± SEM of gene expression (RPKM) of the indicated cluster (D) per developmental time point. Cluster 1 (E) = 13 genes, cluster 2 (F) = 2 genes, cluster 3 (G) = 10 genes, cluster 4 (H) = 8 genes, and cluster 5 (I) = 9 genes.J
Overlap between gene clusters and staged embryonic data from Chen *et al* (2013) shows that cluster 1 mostly consists of not pol II paused pre‐MBT genes.

Since the *w* ^*m4h*^ locus is known to be controlled by classical H3K9me3‐dependent mechanisms, we hypothesized that the upregulation of miRNA would result in the downregulation of factors controlling the epigenetic state at this locus. Differential expression analysis showed that heat shock significantly altered the expression of several mRNAs (Fig 4A; Dataset EV4; 1,136 up vs. 459 down). We could, however, not detect any significant changes in the expression of H3K9me3‐related epigenetic enzymes such as Su(var)3–9, HP1, Eggless (SETDB1), or ATF‐2 (Fig 4B). When comparing all differentially expressed genes with *Drosophila* genes annotated with the GO‐term “heterochromatin formation” (GO:00315007), we found only two downregulated genes, pgc and Elba1 (Fig 4C). In line with this, a GO‐term enrichment analysis of up‐ or downregulated genes did not show an enrichment of epigenetic factors (Appendix Fig S4A and B).

**Figure 5:**
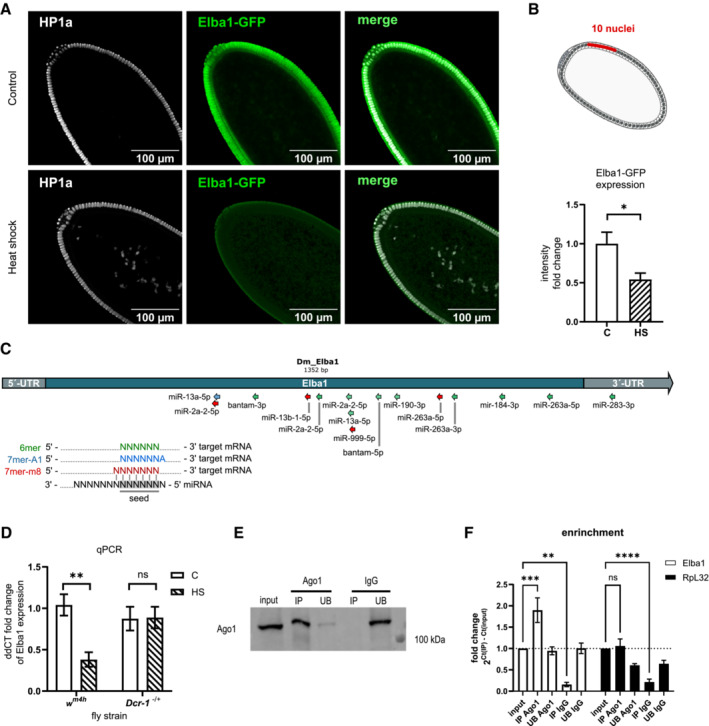
Heat shock‐induced downregulation of Elba1 is dependent on functional miRNA biogenesis Representative confocal images showing stage 5 Elba1‐GFP embryo exposed or not exposed to heat shock during the sensitive developmental period. Green—Elba1‐GFP, White—HP1a. The scale bar represents 100 μm.(Top) Illustrative image of a stage 5 embryo where the red line illustrates the area covering 10 nuclei used for quantification. (Bottom) Quantification of Elba1‐GFP expression using confocal microscopy. *n* = 8 controls and *n* = 5 heat‐shocked samples were quantified, Bar graphs are mean ± SEM, *P = 0.0186 with an unpaired two‐tailed Mann–Whitney test.
*In silico* analysis of potential miRNA‐binding sites on the Elba1 transcript using miRNA seed sequences. Elba1 sequence was downloaded from FlyBase with transcript ID FBtr0077423, and the seed sequences of candidate miRNA were obtained from TargetScan Fly 7.2. Complementary seed sequences were identified using TargetScan Fly's script for the identification of conserved and nonconserved target sites on a custom set of data. Seed matches are reported for miRNAs that were found to be upregulated following heat shock showing a strong inverse correlation to Elba1 (Pearson's *r* < −0.5). Red arrow: 7mer‐m8 match (seed + position 8), blue arrow: 7mer‐A1 match (seed + adenine at position 1 in mRNA), green arrow: 6mer match (seed).
*Elba1* expression measured by qPCR in *w* ^*m4h*^and *Dicer‐1* mutant stage 5 embryos, with and without heat shock. Results are the mean of the fold change of ddCT values ± SEM. *n* (same order as data) = 5, 6, 8, 7 biological replicates, **P = 0.0018 with an unpaired two‐tailed *t*‐test.Representative western blot image demonstrating the presence of Ago1 in a sample of 1,000 *w* ^*1118*^ *Drosophila* stage 5 embryos immunoprecipitated with Ago1 and not with IgG control antibody. IP = immunoprecipitation, UB = unbound.qPCR analysis of Ago1 immunoprecipitated samples. Elba1, but not RpL32, is enriched after Ago1 IP but not after IgG IP. Bar graphs are mean ± SEM, **P = 0.0005, ***P = 0.0011, ****P < 0.0001 using ordinary one‐way ANOVA with the Dunnett's multiple comparison test *n* = 8 biological replicates.

Unsupervised correlation and clustering analysis between the upregulated miRNA and downregulated long RNA revealed a cluster of strong inverse correlation (Fig 4D, cluster 1; Dataset EV5). This cluster consisted of 11 miRNA, including mir‐190, mir‐2a and b, and bantam, and 13 mRNA. Using modENCODEs temporal expression data for all genes in each RNA cluster (Fig 4E–I) we found that our identified gene cluster displays a distinct temporal expression (Fig 4E). To get further insight into this cluster, we compared it with carefully categorized pre‐MBT genes from Chen *et al* (2013) and found that 9 out of 13 genes in this cluster overlapped with nonpaused pre‐midblastula transition (pre‐MBT) genes (Fig 4J). It is interesting to note that genes from the identified pre‐MBT gene cluster have their dominant expression precisely overlapping the end of the stress‐sensitive period (Figs 1C and D, and 4E). Stress‐induced downregulation of such temporally expressed genes could explain why stress at this time, but not after, would modulate variegation of the *w* ^*m4h*^ locus. We therefore investigated this cluster with 13 genes in more detail. Functionally, we found no GO‐term enrichment for this cluster, and only one of them, Elba1, has been reported to be involved in chromatin silencing (Appendix Fig S4C). More specifically, Elba1 has been shown to be a transcriptional repressor and insulator‐binding protein that works in concert with Elba2, Elba3, and Insv to ensure that there is no leakage between transcriptional units (Aoki *et al*, 2012; Ueberschär *et al*, 2019). To verify our findings from the RNA‐seq data, we repeated the heat shock intervention during the sensitive period in embryos expressing Elba1‐GFP. This time, we collected heat‐shocked and control embryos 3–3.5 h after egg laying for fixation and staining with GFP and HP1a. Late stage 5 embryos were carefully staged using a confocal microscope with HP1a as a guide (Fig 5A). We next quantified the Elba1‐GFP expression in 10 peripheral cells per embryo and, in alignment with the RNA‐seq data, we found that a pre‐MBT heat shock significantly reduced Elba1 at embryonic stage 5 (Fig 5B).

To find potential miRNA seed sequences complementary to the Elba1 transcript, we conducted an *in silico* analysis using TargetScan Fly's script (Agarwal *et al*, 2018). We found three types of target sites (7mer‐m8, 7mer‐A1, and 6mer) and identified miR‐283‐3p as having a candidate targeting 6mer to the 3′UTR (Fig 5C). Aside from miR‐283‐3p, we identified several miRNAs with candidate targeting sites in the CDS. Similar to mammals, miRNA targeting to the 3′UTR is most effective, but targeting sites within the CDS also have some silencing effects (Agarwal *et al*, 2018). While multiple miRNA candidates presumably have some silencing effect on Elba1, miR‐283‐3p remains the primary candidate. This is supported by its highest negative correlation to the Elba1 transcript in our earlier correlation analysis.

To experimentally test the causal relationship between heat shock‐induced miRNA and downregulation of Elba1 (Fig 4D; Appendix Fig S5), we used qPCR to compare the Elba1 expression in stage 5 embryos of control and heat‐shocked *w* ^*m4h*^ and *Dicer‐1* (*Dcr‐1*) mutants. *Dcr‐1* is essential and specific for the miRNA synthesis, with minimal impact on the synthesis of other sncRNA (Lee *et al*, 2004). As before, a short 30 min heat shock reduced the *Elba1* expression in *w* ^*m4h*^ flies (Fig 5D). In *Dcr‐1*‐mutant embryos, however, this downregulation was not detectable (Fig 5D). Thus, we concluded that the heat shock‐induced downregulation of *Elba1* relies on a functional miRNA‐processing pathway. To directly test whether the Ago1‐miRNA RISC complex binds Elba1, we performed an Ago1 immunoprecipitation followed by qPCR using an IgG antibody as negative control (Fig 5E and F; Appendix Fig S6). In line with our previous results, we detected an enrichment of Elba1 in the Ago1 IP fraction compared with input, the Ago1 unbound fraction (UB), and the IgG IP control (Fig 5F).

### The insulator‐binding factor Elba binds to de‐repressed genes and acts as a Su(var) for *w* ^*m4h*^

**Figure 6:**
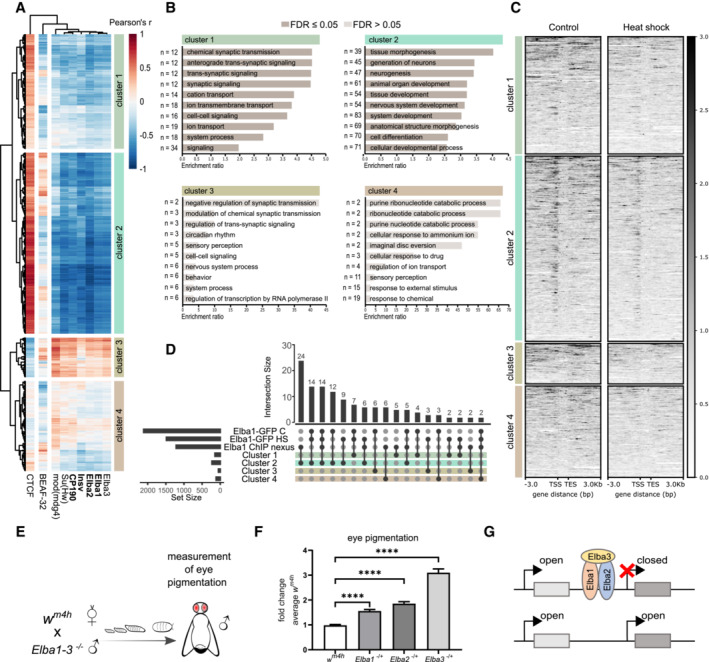
Elba1 binds to specific heat shock‐induced developmental genes and acts as a Su(var) for *w* ^*m4h*^ Correlations (Pearson's *r*) between insulating binding factors and all heat shock‐induced genes with a fold change > 1 (Fig 5A) from stage 5 *w* ^*m4h*^ *Drosophila* embryos. Euclidean clustering shows that cluster 2 correlates inversely with most insulator‐binding factors. *n* = 24 embryos per condition.Gene ontology enrichment analysis of gene clusters from (A) using WebGestalt. The top 10 hits from over‐representation analysis (ORA) for biological processes are presented per cluster.CUT&RUN peak scores of Elba1‐GFP stage 5 embryos centered on genes from clusters in (A). Cluster 2 shows Elba1 enrichment at the TSS regions, which is more pronounced in control than that in heat‐shocked samples. Elba1 enrichment is not visible at TSS regions in the other clusters. Peak scores are based on 5 merged samples of 20 embryos each per condition.Intersection of genes in clusters identified in (A), genes associated with consensus peaks from control and heat shock CUT&RUN samples, and genes identified in Ueberschär *et al* (2019) to be associated with binding sites for Elba1. The graph shows only intersections with ≥ 2 genes. Consensus peaks are based on called peaks from ≥ 2 samples (of 5) per condition.Virgin *w* ^*m4h*^ females were crossed with *Elba1‐3* homozygous mutant males, after which eye pigmentation was measured in 5‐day‐old adult male offspring.Eye pigmentation in *w* ^*m4h*^ and *Elba1‐3* heterozygous males. Results are mean ± SEM of *n* = 58 controls, *n* = 51 *Elba1* ‐, *n* = 19 *Elba2* ‐, and *n* = 13 *Elba3* heterozygous mutant samples. ****P < 0.0001 using a two‐tailed *t*‐test.Schematic model of how the Elba insulator‐binding complex suppresses position‐effect variegation in *w* ^*m4h*^ by a partition of chromatin states and gene expression.

Considering that the Elba complex is important to minimize transcriptional leakage (Aoki *et al*, 2012; Ueberschär *et al*, 2019), we reasoned that the heat shock‐induced upregulation of genes (Fig 4A) might be a consequence of reduced levels of Elba1. Unsupervised cluster analysis of Pearson's *r* scores between insulator‐binding factors and heat shock‐induced genes revealed four gene clusters of which cluster 2, the largest cluster, had a strong negative correlation to Elba1, Elba2, Insv, and CP190 (Pearson's *r*: Elba1 mean = −0.66, SD = 0.10; Elba2 mean = −0.76, SD = 0.11; Insv mean = −0.68, SD = 0.10 and CP190 mean = −0.63, SD = 0.11; Fig 6A, cluster 2; Dataset EV6). Functional analysis of clusters showed an over‐representation of genes involved in developmental and morphological progress in cluster 2 (Fig 6B, top right). As developmental and morphological associated genes were identified by Ueberschär *et al* (2019) to be controlled by the Elba complex, we compared our data with their ChIP‐data for Elba1‐3 and Insv (Appendix Fig S7A–E). In agreement with the loss of Elba1‐restricted transcription, we found an overlap between genes in cluster 2 and genes associated with Elba1‐3 ChIP‐peaks.

**Figure EV3:**
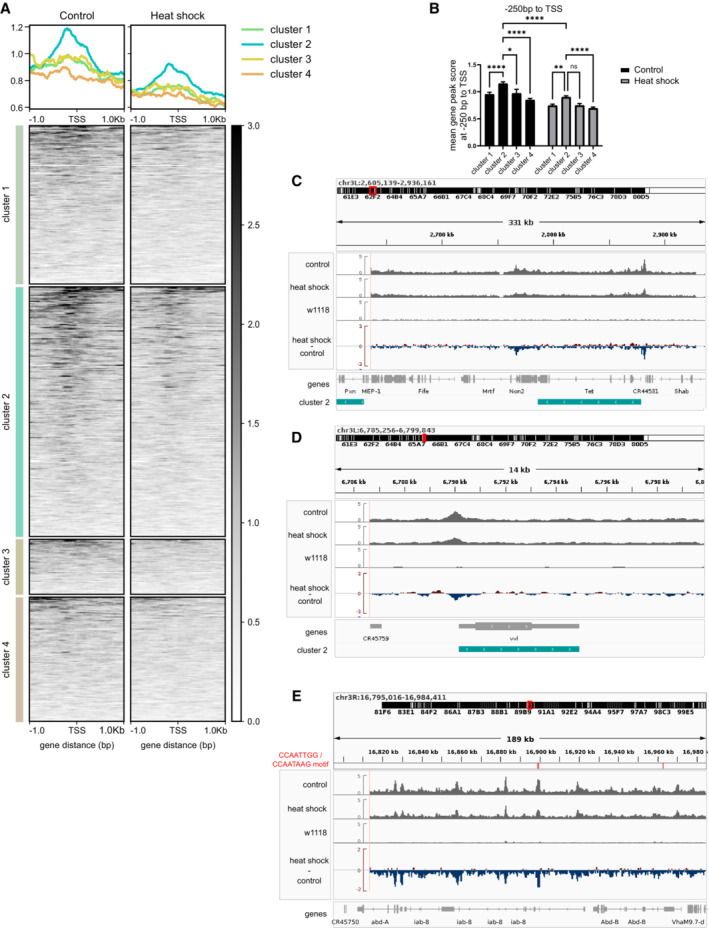
CUT&RUN tracks of Elba1‐GFP in *Drosophila* embryo at representative loci CUT&RUN was made using a GFP antibody on Elba1‐GFP or *w* ^*1118*^ stage 5 embryos. The Elba1‐GFP embryos were either exposed to 30 min heat shock pre‐MBT, or kept as controls.A
Heatmap and profiles of peak scores centered over TSS at genes from clusters 1–4 (Fig 6A). Peak scores are based on 5 merged samples of 20 embryos each per condition.B
Quantification of mean gene peak score per indicated gene cluster (Fig 6A) at 250 bp upstream TSS to TSS. Cluster 2 shows increased Elba1 binding at the TSS compared with other gene clusters, and this binding is higher in control than in heat‐shocked embryos. Results are mean ± SEM, *P = 0.0314, **P = 0.0022, ****P < 0.0001 using two‐way ANOVA with the Šídák's multiple comparisons test. Peak scores are based on 5 merged samples of 20 embryos each per condition.C, D
CUT&RUN tracks showing two representative loci on chromosome 3 together with heat shock‐induced genes from cluster 2 in Fig 6A.E
CUT&RUN peaks showing the Fab‐7 region of the bithorax complex, which is a well‐known Elba‐binding site. The Elba binding motifs are marked in red. All tracks are merged per condition *n* = 5 (controls and heat shock), *n* = 2 *w* ^*1118*^.

To test whether an early heat shock decreases the Elba1 binding to these genes, we performed CUT&RUN using GFP antibody on five sets of 20 stage 5 Elba1‐GFP embryos exposed to either 30 min heat shock during the sensitive period or kept as controls. As we had little starting material, we merged the 5 replicates within each experimental group (individual datasets are available under Data availability). Looking at the peak scores associated with these gene regions, we detected increased Elba1 binding at the TSS region of cluster 2 genes, compared with genes from the other clusters (Figs 6C and EV3A and B). Heat‐shocked embryos showed reduced peak scores at the TSS region of cluster 2 genes, despite having similar profiles (Figs 6C and EV3A and B). CUT&RUN tracks over representative genomic loci are found in Fig EV3C–E. We further aligned the consensus peaks and compared them to all upregulated genes. As before, the greatest overlap was detected between Elba1‐GFP peaks and cluster 2 (Fig 6D).

**Figure EV4:**
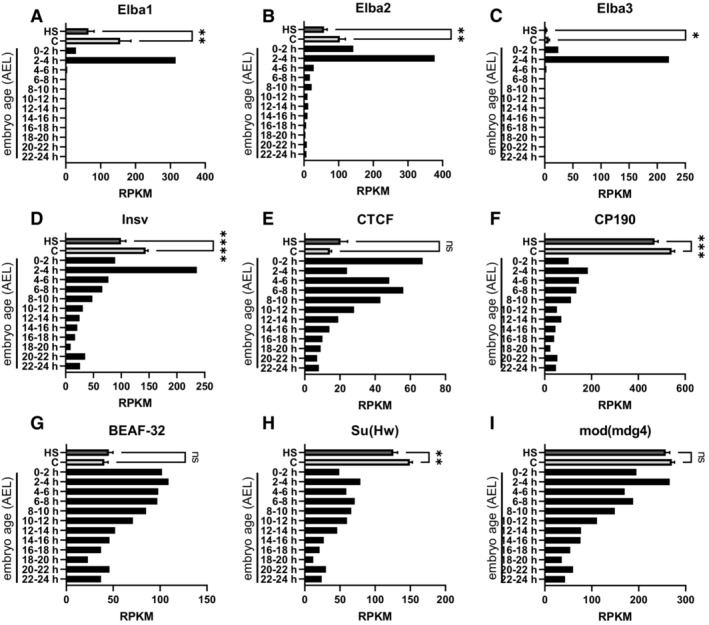
Embryonic expression of insulator‐binding factors Expression levels of known insulator‐binding factors at different embryonic ages and in control or heat‐shocked stage 5 embryos. Data were extracted from modENCODE, AEL = after egg laying. C = control and HS = heat‐shocked *w* ^*m4h*^ stage 5 embryos. ns = nonsignificant. *n* = 24 single embryos per condition.A–C
Elba factors are specifically expressed (especially Elba1 and Elba3) during pre‐MBT and MBT. Error bars represents mean ± SEM, Elba1 *P* (**) = 0.0014, Elba2 *P* (**) = 0.0094, Elba3 *P* (*) = 0.0128, using an unpaired two‐tailed Mann–Whitney test.D
Insv is highly expressed in 2‐ to 4‐h‐old embryos but continue to be expressed throughout embryogenesis. Error bars represent mean ± SEM, *P* (****) ≤ 0.0001 using an unpaired two‐tailed *t*‐test.E–I
The other factors are also expressed throughout embryonic development but with a declining trend. Error bars represents mean ± SEM, CP190 *P* (***) = 0.0009, Su(Hw) *P* (**) = 0.0017 using an unpaired two‐tailed *t*‐test.

Since not only Elba1 but also Elba2 and Elba3 showed a similar clustering (Fig 6A), we next looked more closely at insulator‐binding factors with different temporal expression profiles during embryogenesis (Fig EV4). We analyzed their expression between the heat‐shocked and control embryos and found that several insulating binding factors were statistically significantly downregulated following heat shock (Fig EV4). Most interestingly, we found that the expression of all members of the Elba complex and Insv was reduced, although not enough (with the exception of Elba1) to reach the Log_2_ fold change threshold of < −1 or the FDR used when analyzing the whole dataset.

To test whether the reduced expression of the Elba complex during MBT would result in long‐term epigenetic effects in the adult fly, we next crossed female virgins from the *w* ^*m4h*^ PEV‐strain with homozygous *Elba1‐3* mutant males (Fig 6E). Intriguingly, eye pigmentation of 5‐day‐old males showed loss of *white* silencing in all Elba heterozygous mutants, thus mimicking the effect of an early embryonic heat shock (Fig 6F and G). In all, our study points to a central role for miRNA‐Elba dependent fine‐tuning of the emerging chromatin landscape, that—if disrupted—may have life‐long consequences on the phenotype.

## Discussion

Here, we provide new insights into the dynamics of sncRNA during the earliest stages of *Drosophila* embryogenesis and their response to heat shock. We found that heat shock induced an extensive increase in maternal miRNA, and by combining transcriptome‐wide data of both sncRNA and long RNA from the same single embryos, we revealed a strong association between heat shock‐induced upregulation of a specific group of miRNA (e.g., mir‐13, mir‐2, and bantam) and reduction in a gene cluster consisting of pre‐MBT genes. One of these genes, a newly described insulator‐binding factor—Elba1, acts as a transcriptional repressor to ensure correct gene expression during early development (Ueberschär *et al*, 2019). In line with this function, we found that heat shock in the first hour of embryogenesis results in an upregulation of genes involved in developmental patterning. These upregulated genes showed a substantial overlap with ChIP‐peaks for Elba1‐3 and CUT&RUN peaks for Elba1‐GFP. Most important, we found that heat shock reduced such peaks. Moreover, the heat shock‐induced downregulation of Elba1 was attenuated in *Dcr‐1* mutant embryos, and Elba1 transcript was found to be bound to Ago1. Finally, the reduction in the components of the Elba complex efficiently mimicked the original effect on *w* ^*m4h*^ caused by the embryonic heat shock. Thus, our results suggest a miRNA‐driven control of the zygote's first transcriptome to set the tone for forthcoming gene expression.

It has earlier been reported that there is a maternal deposit of miRNA in *Drosophila* eggs (Marco, 2015). From our data, it is clear that several of these maternal miRNAs (e.g., mir‐14, mir‐999, mir‐92b, and bantam) show a declining trend during the first 5 stages of embryogenesis, and that a heat shock attenuates their degradation. The degradation of maternal miRNA is not as well understood as the degradation of maternal mRNA but has been proposed to be controlled via 3′‐end adenylation by the noncanonical poly(A) polymerase Wispy (Lee *et al*, 2014). If the retained maternal miRNA we detect in response to heat shock is controlled by Wispy or some other pathway remains, however, to be tested.

While our experiment with *Dcr‐1* reveals a dependence on the miRNA machinery in regulating the heat shock‐induced downregulation of *Elba1*, it does not distinguish between maternal and zygotic miRNA. Unsupervised clustering, however, separates maternal and zygotic miRNAs into different clusters of correlation. In this analysis, the maternal miRNAs showed the highest negative correlation with the identified pre‐MBT genes, suggesting a more dominant role for the maternal miRNA for their regulation. It is, however, common that miRNAs have overlapping and redundant functions (Fu *et al*, 2014), and this is likely also the case for miRNA‐controlled gene regulation in the early embryo.

Curiously Elba1 does not have any homologs, and this is not specific to Elba1 but a general feature of the earliest transcribed genes. They are often short, newly evolved, and differ across species (Heyn *et al*, 2014). Moreover, even though they code for nucleic acid‐binding and zinc‐binding proteins, as well as sequence‐specific DNA‐binding transcription factors, they are most often nonessential genes. Rather, it has been proposed that the species differences during the MBT have created opportunities for the evolution of new genes that can modulate the zygotic gene program (Heyn *et al*, 2014). The fact that Elba mutant flies are perfectly viable in combination with our findings that their amplitude is determined by stress further reinforces the notion that they are nonessential modulators of early zygotic transcription.

The position‐effect variegation strain *w* ^*m4h*^ has been extensively used for epigenetic research and enabled the discovery of multiple Su(var)s and E(var)s. (Phalke *et al*, 2009). Since there is the same, or similar, degree of variegation on both eyes, it has been concluded that the variegation must be set very early in development and then maintained up to adulthood (Bughio *et al*, 2019). We found, as shown before, that the most sensitive period to modulate the variegation of the *w* ^*m4h*^ strain is before the MBT (Hartmann‐Goldstein, 1967; Lu *et al*, 1998; Seong *et al*, 2011; Bughio *et al*, 2019). Considering what we know about the *de novo* formation versus maintenance of heterochromatin (Allshire & Madhani, 2018), it might not be so surprising that the developmental window just before the *de novo* heterochromatin formation is a sensitive period, whereas after is not.

The three members of the Elba family have a very peculiar, very short temporal expression just before the time for *de novo* heterochromatin formation (Fig EV4; Singer & Lengyel, 1997). They have been reported to work as transcriptional repressors and insulator‐binding factors that ensure proper partitioning of transcriptional units during early embryogenesis (Dai *et al*, 2015; Ueberschär *et al*, 2019). We report here that their expression, although restricted to a brief period, will have a long‐lasting effect on the adult heterochromatin. More specifically, we find them all to be Su(var)s for *w* ^*m4h*^. We can, at this point, only speculate how the Elba family of proteins might influence the variegation of *w* ^*m4h*^. First, since Elba 3 contains a PxVxL motif that suggests it to be a binding partner to HP1a (Meyer‐Nava *et al*, 2020), it might have a direct role in the recruitment of HP1 to the chromatin. Second, since insulator‐binding factors have a pivotal role during the *de novo* heterochromatin formation it is possible that the Elba complex with or without HP1 has a role in setting up borders.

## Materials and Methods

### Fly husbandry

The *ln(1)w* ^*m4h*^ *Drosophila* strain (Muller, 1930) was kindly provided from Gunter Reuter's lab and has been maintained in a climate‐controlled 22°C incubator and kept on standardized food. Flies used for experiments were inbred for > 10 generations and flies with complete loss of PEV were not used for crossing to enable capture of differences of variegation. *Dicer‐1* ^Q1147X^ mutants (Lee *et al*, 2004; #RRID:BDSC_32066) containing a nonsense codon at the PAZ domain were kept at 22°C for > 6 generations on standard food before egg collection. *Elba1* sk6, *Elba2* sk2, and *Elba3* sk5 mutant flies homozygously carrying the respective frame‐shift mutation were kindly provided from Qi Dai's lab (fly strains are described in Ueberschär *et al*, 2019) and kept in room temperature (at approximately 22°C) on standardized food. *Elba1‐3* ^−/−^ − *ln(1)w* ^*m4h*^ crossings were kept in a climate‐controlled 22°C incubator on standardized food. The Elba1‐GFP fly strain (RRID:BDSC_83657) and *w* ^*1118*^ were kept in a 26°C incubator on standardized brown food.

### Eye pigment measurement

For screening of sensitive periods: eggs were collected on juice agar plates in tight intervals (30 min—1 h) and exposed to one heat shock session in a 37°C incubator, or were kept as controls. The selection was random. For PEV expression in *Elba1* mutants: virgin *ln(1)w* ^*m4h*^ were crossed with either *Elba1‐3* mutant males or *ln(1)w* ^*m4h*^ males and left to mate and lay eggs. Five or six different vials per crossing were set up and all were flipped 3 times. All experiments: flies were left to develop in a climate‐controlled 22°C incubator. Males were decapitated 5 days after eclosure and their heads, collected in groups of 3–10, were first frozen in liquid nitrogen and then homogenized with a 5 mm ∅ metal bead (Qiagen) for 2 min at 40 Hz using TissueLyser LT (Qiagen). 500 μl PBS‐tween (0.01%) was added and samples were shaken, kept at room temperature for 1 h, and centrifuged. Absorbance at A480 was measured on supernatant in technical doublets using VersaMax (Molecular Devices) microplate reader. *ln(1)w* ^*m4h*^ heat shock experiments: At least two biological replicates were collected per heat shock time and experiment, and heat shock experiments were performed 7 times. *Elba1‐3* mutants—*ln(1)w* ^*m4h*^ experiments: 6–10 heads were analyzed per sample. *n* = 58 *w* ^*m4h*^ × *w* ^*m4h*^‐, *n* = 51 *Elba1* ^*SK6*^ × *w* ^*m4h*^‐, *n* = 19 *Elba2* ^*SK2*^ × *w* ^*m4h*^‐, and *n* = 13 *Elba3* ^*SK5*^ × *w* ^*m4h*^ crossings were collected and analyzed.

### Sampling for sncRNA sequencing of developmental timeline

Eggs were collected on juice agar plates for 30 min and were immediately dechorionated. The staging was performed under SMZ 745 (Nikon) microscope using the criteria for Bownes' stages 1–5 (Bownes, 1975). Single embryos were collected in 2 μl RNase‐free water with Recombinant RNase inhibitor (TAKARA) and ruptured with an RNase‐free needle. One 5 mm ∅ metal bead (Qiagen) and 500 μl Qiazol (Qiagen) were added per sample and the samples were homogenized for 2 min at 40 Hz using TissueLyser LT (Qiagen). *n* = 5 of stage 1–3 and *n* = 4 of stage 4 and 5.

### Sampling for sncRNA and long RNA sequencing after exposure to heat shock

Eggs were collected on juice agar plates in 30 min intervals and immediately exposed to one session of heat shock at 37°C for 30 min or kept as controls. This selection was random. Embryos were thereafter kept in a climate‐controlled 22°C incubator for approximately 2 h, dechorionated in 3.5% bleach, and staged under SMZ 745 (Nikon) bright‐field microscope using the criteria for Bownes' stage 5 (Bownes, 1975), including formed cells at egg surface and round pole cells at the posterior axis. Single embryos were collected in 2 μl RNase‐free water with an RNase inhibitor and ruptured with an RNase‐free needle. One 5 mm ∅ metal bead (Qiagen) and 500 μl Qiazol (Qiagen) were added per sample and the samples were homogenized for 2 min at 40 Hz using TissueLyser LT (Qiagen). *n* = 24 single embryos per condition.

### RNA extraction and small RNA library preparation

RNAs were extracted using miRNeasy Micro Kit (Qiagen) according to manufacture protocol. Quality was confirmed using Agilent RNA 6000 Nano kit (Agilent) on the 2100 Bioanalyzer Instrument (Agilent) prior to storage at −70°C. NEBNext Small RNA Library Prep Set for Illumina (New England Biolabs) was used for library preparation according to the manufacturer's protocol with some changes. We downscaled all samples to half volume and added 2S rRNA block oligo (5′‐TAC AAC CCT CAA CCA TAT GTA GTC CAA GCA‐SpcC3 3′; 10 μM; Wickersheim & Blumenstiel, 2013) to a final concentration of 2.5 μM together with SR‐RT primer (from the kit). Primers and adaptors from the kit were diluted 1:4 until PCR amplification, according to starting RNA concentration. PCR amplification was run for 15 cycles and NEBNext Index1‐24 primers for Illumina were used (New England Biolabs).

Libraries were cleaned using Agencourt AMPure XP (Beckman Coulter) and run on precasted 6% polyacrylamide Novex TBE gel (Invitrogen). Bands of sizes 140–170 bp were selected. Gel extraction was made by centrifugation at 15,000 *g* using gel breaker tubes (IST Engineering Inc) in DNA Gel Elution Buffer provided in the NEBNext kit. Samples were incubated at 37°C for 1 h on a shaker, frozen at −70°C for 15 min, and incubated at 37°C on a shaker for 1 h once more. Gel debris was removed by Spin‐X 0.45 μm tube. Libraries were precipitated overnight at −70°C in 1 μl GlycoBlue (Invitrogen), 0.1 × volume of 3 M acetate (pH 5.5), and 3 × volume of 100% ethanol. Library sizes were measured on 2100 Bioanalyzer instrument (Agilent) using the Agilent High Sensitivity DNA kit (Agilent) and concentration was determined using QuantiFluor ONE ds DNAsystem on Quantus fluorometer (Promega). Equal concentrations of libraries were pooled and sequenced on NextSeq 500 sequencer using NextSeq 500/550 High Output Kit v2 with 75 cycles (Illumina). Unique sample IDs are summarized in Dataset EV8.

### Preprocessing of sncRNA‐sequencing results

We used Cutadapt version 1.18 (Martin, 2011) to trim the adaptor sequence (AGATCGGAAGAGCACACGTCTGAACTCCAGTCACAT) from sncRNA reads and FastQC v.0.11.5 (Andrews, 2015) for quality filtering. Reads between 14‐ and 80 nucleotides containing the adaptor and with more than 80% of the bases having a phred quality score (Q‐score) > 20 were retained. Mean sequence depth was 18.06 M reads (min = 12.01 M, max = 38.36 M) for the stage 1–5 data and 15.98 M reads (min = 12.15 M, max = 20.21 M) for the heat shock experiment data.

Trimmed reads were further mapped using SPORTS pipeline version 1.0.5 (Shi *et al*, 2018) with standard settings except following modifications; we replaced Rfam with repeatmasker (Dataset EV9), which was placed at the bottom of the hierarchy. Within this pipeline, Bowtie version 1.1.2 (Langmead *et al*, 2009) was used with the following input; −M 1 ‐‐strata ‐‐best ‐v 1, returning one single read allowing one mismatch. Alignment was performed to the dm6 reference genome and then to sncRNA‐specific references using the following hierarchy; miRNA, tRNA, rRNA, piRNA, other ncRNA, and repeats. For details and annotation sources, see Dataset EV9. The number of reads, read‐length, and annotation hits were retained per unique sequence.

### sncRNA‐seq analysis

A list containing experimental metadata, annotation information, and count table was retained and filtered; minimum of 20 reads per sequence in 17% of samples for stage 1–5 timeline experiment and minimum of 20 reads per sequence in 50% of samples for heat shock experiment. An additional filter removing sequences with less than 0.01 rpm per sequence (in 17% of samples for the timeline experiment or 100% of samples for heat shock experiment) was applied and sequences were assigned to a sncRNA class according to regular expressions retained from the annotation hits. Differential expression analysis was performed in DEseq2 (version 1.24.00) with the design ~ Intervention + flow cell run. We used Euclidean clustering within the pheatmap package (version 1.0.12) and a cutoff at Log2 ± 1 based on stage 5 vs. stage 1 differential expression to determine the maternal or zygotic origin of miRNAs.

### Long RNA library preparation

DNA was digested from aliquots from the same RNA extracted as described above (RNA extraction and smallRNA library preparation) with RNase‐Free DNase Set (Qiagen) according to kit protocol and concentrated using Oligo Clean & Concentrator (Zymo Research) according to kit protocol but adjusted for sample volumes. RNA quality was determined on the 2100 Bioanalyzer Instrument (Agilent) using RNA 6000 Nano kit (Agilent).

cDNA was synthesized using Ovation RNA‐Seq Systems 1–16 for model organisms (NuGEN) according to the kit protocol. Samples were sonicated 6 times in 15 s on‐ 15 s off‐intervals using the Bioruptor Pico sonication device (diagenode). Library construction was done using the Ovation RNA‐Seq Systems 1–16 for model organisms (NuGEN) according to protocol, and cycles for library amplification were determined using 7900HT Fast Real‐Time PCR System (Applied Biosystems™). The amplification buffer and enzyme mixes provided in the library kit were used for the master mix together with EvaGreen for qPCR (Biotium). Libraries were amplified according to the mean cycle for exponential PCR amplification per experiment (16 cycles) and purified according to protocol. Library sizes were measured on the 2100 Bioanalyzer Instrument (Agilent) using the High Sensitivity DNA chip (Agilent) and concentrations were determined using QuantiFluor ONE ds DNAsystem on Quantus fluorometer (Promega). Equal concentrations of libraries were pooled and sequenced on the NextSeq 500 sequencer using NextSeq 500/550 High Output Kit v2 with 75 cycles (Illumina). Unique sample IDs are summarized in Dataset EV8.

### Preprocessing and analysis of long RNA‐sequencing results

We used Cutadapt version 1.18 (Martin, 2011) to trim the adaptor sequence (AGATCGGAAGAGCACACGTC) from long RNA reads and FastQC v.0.11.5 (Andrews, 2015) for quality filtering. Reads over 14 nucleotides and with more than 80% of the bases having a phred quality score (Q‐score) > 20 were retained. Depth per library was 21.54 M reads (min = 20.26 M, max = 31.66 M reads). STAR genome index files were generated using Drosophila_melanogaster.BDGP6.28.dna.toplevel fasta and Drosophila_melanogaster.BDGP6.28.101.gtf (Ensembl). These genome index files were then used on trimmed reads using STAR (v.2.5.0a; Dobin *et al*, 2013) with standard settings and indexed using SAMtools (v.1.3.1; Li *et al*, 2009a). Standard featureCounts (v.1.5.0‐p1; Liao *et al*, 2014) settings were used for assigning reads to genomic features, with minimal overlap of 15 bases. A list containing experimental metadata, annotation information, and count table was retained and filtered (minimum 10 counts per read in 50% of samples).

To compare maternally loaded and early zygotic genes between control and heat‐shocked embryos, we extracted the genes in our data that matched the classifications made by Lott *et al* (dataset S1 in Lott *et al*, 2011; maternal) or by De Renzis *et al* (table S8 in De Renzis *et al*, 2007; early zygotic). We further used the expression profile of sub‐stages of nuclear cycle 14 from dataset S1 in (Lott *et al*, 2011) to differentiate nuclear cycle 14 expressed genes. The web‐based gene set analysis toolkit (WebGestalt; Wang *et al*, 2017) was used for functional analysis using the over‐representation analysis of biological processes with the BH FDR method. To generate matrixes of correlation, we used the rcorr function with the Pearson's option within the Hmisc package (version 4.2‐0) and Euclidean clustering within the pheatmap package (version 1.0.12). For analysis of overlaps with pre‐MBT classifications, we used classifications made by Chen *et al* (table 1 in Chen *et al*, 2013), to compare against genes from indicated gene clusters.

### Quantitative real‐time PCR

Eggs from *w* ^*m4h*^, *Dcr‐1*‐ and *Elba1* mutants were collected on juice agar plates for 1 h and immediately heat‐shocked for 30 min (as earlier described) or kept as control. All samples were dechorionated in 3.5% bleach and staged under an SMZ 745 (Nikon) microscope as described above. 2–5 stage 5 embryos were collected per sample in 4 μl RNase‐free water with RNase inhibitor and ruptured with an RNase‐free needle. One 5 mm ∅ metal bead (Qiagen) and 500 μl Qiazol (Qiagen) were added per sample and shaken for 2 min at 40 Hz using TissueLyser LT (Qiagen). RNAs were extracted using miRNeasy Micro Kit (Qiagen) according to manufacture protocol and good quality was confirmed using Agilent RNA 6000 Nano kit (Agilent) on the 2100 Bioanalyzer Instrument (Agilent). iScript cDNA Synthesis Kit (BIO‐RAD) was used for cDNA synthesis and a master mix of iTaq Universal SYBR Green Supermix (BIO‐RAD), RNase‐free H_2_O, and primers (Merch) was prepared according to manufacturer's protocols. Samples and master mix were loaded in triplicates onto 96 well plates and read on a 7500 Fast Real‐Time PCR system (Thermo Fisher Scientific). Primers used: *Elba1* forward: TGTCCTTAGCAGCTTCTCAG, reverse: CGCATTCAAGATGCAAATGAG. *Dicer‐1* forward: AGGAGACAAAGCGGGCAAAG, reverse: TATGCGGTACAGGATGCAGG. *RpL32* (for normalization) forward: CCGCTTCAAGGGACAGTATC, reverse: ACGTTGTGCACCAGGAACTT. Relative expression toward *w* ^*m4h*^ control embryos was analyzed using the ΔΔCT method.

### Immunoprecipitation

*W* ^*1118*^ *Drosophila* embryos were collected in 1 h intervals, aged for 1–1.5 h, dechorionated in 3.5% bleach, and washed with RNase‐free H_2_0. Embryos were put in RNase‐free PBS and transferred in batches of 100–500 to Eppendorf tubes where the PBS was discarded. PBS with cOmplete Mini EDTA‐free protease inhibitor cocktail (Sigma) and RNase inhibitor was added to each batch and the samples were snap frozen. 1,000 embryos were pooled into one tube (*n* = 8), the PBS was removed and lysis buffer (50 nM Tris (pH 7.5), 100 mM KCl, 12 mM MgCl_2_ 1% Nonidet P‐40, 1 mM DTT, 100 μg/ml Cyclohexamide, 1× cOmplete Mini EDTA‐free protease inhibitor cocktail and 1 μl/ml RNase inhibitor) was added. The samples were homogenized using the “tight” Dounce Tissue Grinder and centrifuged at 4°C for 10 min at 10,000 *g*. The supernatants were collected and 10% of each sample was collected as input. Of each, one half was used for RNA extraction and 400 μl Qiazol was added and the samples snap frozen on dry ice. For the other half, 1× NuPAGE LDS Sample Buffer (Invitrogen) and 0.5 μl 2‐mercaptoethanol were added. Protein samples were boiled at 80°C for 10 min and put on dry ice.

For immunoprecipitation, the embryo lysates were initially precleared using Dynabeads™ Protein G (Invitrogen) for 30 min at 4°C. The lysates were divided in two and incubated with rabbit anti‐Ago1 polyclonal (Abcam, ab5070) or rabbit IgG polyclonal (Abcam, 171870) on rotation at 4°C overnight followed by incubation with Protein G‐dyna beads for 2–4 h at 4°C on rotation. Unbound sample (UB) was collected and prepared for RNA or protein extraction (as described above). The beads were washed in high salt buffers (50 mM Tris (pH 7.5), 300 mM KCl, 12 mM MgCl_2,_ 1% Nonidet P‐40, 1:250 DTT, 0.5 μl/ml RNase inhibitor, 100 μg/ml Cyclohexamide and 1× cOmplete Mini EDTA‐free protease inhibitor cocktail) and extra high salt buffer (high salt buffer +300 nM NaCl). We diluted an aliquot of beads in lysis buffer and used those for western blot (as described below). The rest of the beads were resuspended in 400 μl Qiazol and processed for RNA extraction, quality assessment, and qPCR, as described earlier (see Quantitative Real‐Time PCR). Equal loading of tot RNA was assured by QuantiFluor ONE ds DNAsystem on Quantus fluorometer (Promega). Primers used: *Elba1* forward: TGTCCTTAGCAGCTTCTCAG, reverse: CGCATTCAAGATGCAAATGAG. *RpL32* (reference gene) forward: CCGCTTCAAGGGACAGTATC, reverse: ACGTTGTGCACCAGGAACTT. Enrichment was calculated by 2^−(mean Ct (sample) – mean Ct(sample input))^.

For IP assay control, SDS–PAGE, protein transfer onto PVDF membrane, and western blotting were all performed using standard procedures on the prepared protein extracts. Primary rabbit anti‐Ago1 polyclonal (Abcam, ab5070) antibody in the dilution of 1:1,000 was used, and the proteins detected using the secondary antibody from Li‐COR diluted 1:15,000 (IRdye donkey anti‐rabbit 800CW). Nonspecific proteins were blocked using 4% BSA in PBS with 0.1% tween and all antibody dilutions were made in the same block solution. PBS with 0.1% tween was used as a wash buffer. Membranes were imaged using Odyssey^®^ DLx Imaging System (Li‐COR).

### *In silico* analysis of possible miRNA target sites on Elba1

The Elba1 transcript was downloaded from FlyBase with transcript ID FBtr0077423. The seed sequences of all upregulated miRNA families with a strong negative correlation to Elba1 (Pearson's *r* < −0.5) were obtained from TargetScan Fly release 7.2 (Agarwal *et al*, 2018). The miRNA seed target analysis using the full Elba1 transcript was done using TargetScan Fly's script, targetscan_70.pl.

### Immunostaining

Eggs from Elba1‐GFP flies were collected on juice agar plates for 1 h and heat‐shocked at 37°C for 30 min (as described above) or collected for 2 h and kept at 26°C as controls. Eggs were detached from plates, rinsed, and dechorionated in 3.5% bleach for 3–3.5 h after respective cage setup. Eggs were rinsed in water and fixated for 30 min in a 1:1 solution of 4% PFA and n‐heptane. The PFA layer was removed and an equal volume of 99.9% methanol was added. For removal of the vitelline membrane, the vials were hand‐shaken for ~60 s and moved to new tubes. Eggs were washed several times with 99.9% methanol and stored at −20°C. The samples were stepwise rehydrated in 80/20, 60/40, 40/60, and 20/80 mixture of 99.9% methanol and 0.2% PBT (PBS + Triton X‐100) for 5 min each and then blocked in PBTN (0.2% PBT with 4% horse serum) for 1–3 h RT. The samples were incubated overnight at 4°C with primary antibodies (Rabbit anti‐GFP (Torrey Pines Biolabs # TP401) and mouse anti‐C1A9‐s (1ea; Developmental Studies Hybridoma Bank)) diluted 1:500 with PBTN. Samples were then washed x 4 in PBT and incubated with secondary antibodies (Alexa fluor 488 donkey anti‐rabbit; Life Technologies # A21206) and Rhodamine Red‐X‐conjugated donkey anti‐mouse (Jackson immunofluorescence laboratories # 715‐295‐150) diluted 1:500 with PBTN for 2 h RT. The samples were washed × 4 in PBT and finally mounted on slides in Vectashield Vibrance Antifade Mounting Medium (Vector Laboratories, Inc. Ref: H‐1700). We conducted a pilot trial mounting embryos in Vectashield containing DAPI to facilitate staging. However, the emission spectra from DAPI interfered with the detection of the Alexa fluor 488, and we therefore decided to exclude DAPI from further experiments.

### Confocal microscopy, quantification, and image processing

For reliable quantification, we captured one middle stack intersection of control and heat‐shocked stage 5 embryos at 20x on an LSM700 upright confocal microscope (ZEISS microscopy) using the same gain and acquisition settings between all images. Fijis ImageJ2 (version 1.53f55, java version: 1.8.0_172) rolling ball radius algorithm was used for background subtraction and kept constant between samples. The Elba1‐GFP expression was quantified from 10 adjacent nuclei per embryo, using the freehand line option where the line width was set according to nucleus size. The integrated density was used to calculate the relative expression between the two conditions. For visualization purposes only, the brightness and the contrast for each channel were similarly modified for both samples using Fijis ImageJ2 brightness/contrast function.

### CUT&RUN

Elba1‐GFP and *w* ^*1118*^ eggs were collected on juice agar plates in 45 min intervals and immediately exposed to one session of heat shock at 37°C for 30 min or kept as controls (as described above). Embryos were thereafter kept in a climate‐controlled 22°C incubator for approximately 2 h, dechorionated in 3.5% bleach, and staged under SMZ 745 (Nikon) bright‐field microscope using the criteria for Bownes' stage 5 (Bownes, 1975). 20 embryos were collected in 140 μl nuclear extraction buffer (described in Zambanini *et al*, 2022) and ruptured with an RNase‐free needle. *n* = 5 samples of 20 embryos per condition (Elba1‐GFP), *n* = 2 *w* ^*1118*^ (no GFP‐control) samples. The samples were centrifuged for 10 min 700 *g* at 4°C and the supernatant was discarded. Pellet was resuspended in 100 μl nuclear extraction buffer. Bead‐, primary antibody, and pAG‐MNase binding, digestion, fragment release, beads clean up, library preparation, and gel extraction was made exactly similar to the CUT&RUN low volume‐Urea protocol described in (Zambanini *et al*, 2022) with the addition of adding 0.1 ng/ml CUTANA™ Ecoli spike‐in to the stop buffer mix, and using the rabbit‐anti‐GFP (Abcam, ab290) 1:200. Concentrations were determined using QuantiFluor ONE ds DNAsystem on Quantus fluorometer (Promega) and equal library concentrations were pooled and sequenced (paired‐end) on the NextSeq 500 sequencer using NextSeq 500/550 High Output Kit v2.5 with 75 cycles (Illumina).

### Peak calling and CUT&RUN analysis

Quality control was made using FastQC (v.0.11.5; Andrews, 2015) and adaptor trimming using BBDuk from the BBTools suite (v. 39.01; Bushnell *et al*, 2017). Post trim QC, aligning target and spike‐in, spike‐in and *w* ^*1118*^ GFP‐control normalization, peak calling, and consensus peak reporting were made using nf‐core's CUT&RUN v3 pipeline (Ewels *et al*, 2020), using standard settings, setting the iGenome reference to dm6 (spike‐in: K12‐MG1655), and using the de‐duplication of target. We used macs2 as the primary peak caller and included peaks found in ≥ 2 samples as the threshold for consensus peaks.

As the starting material was low, we merged the normalized bigWig files per experimental condition. These files were used to compute Matrix and plot heatmaps and profiles using deepTools (v. 3.5.1; Ramírez *et al*, 2016), as well as to demonstrate representative genomic areas using IGV (v. 2.14.1). Unique sample files can be found under BioProject: PRJNA729249 (see Data availability). For overlaps between heat shock‐induced upregulated genes, CUT&RUN peaks and Insv and Elba factor‐binding sites, genes notated with corresponding binding sites in Ueberschär *et al* (supplementary data 4 in Ueberschär *et al*, 2019) was extracted. The consensus peaks (Dataset EV7) from the CUT&RUN experiment were aligned to the closest gene using ChIPseeker's (v.1.28.3; Yu *et al*, 2015) annotatePeak function using default values with TxDb.Dmelanogaster.UCSC.dm6.ensGene as reference. UpSetR (v. 1.4.0; Conway *et al*, 2017) was used to illustrate the intersections to the indicated clusters.

### Statistics

All statistical analysis was done in R 3.6.0, R 4.1.0, or GraphPad Prism v.8.4.3 and v.9.1.2. For eye pigment statistical analysis, ordinary one‐way ANOVA with the Dunnett's multiple comparison or two‐tailed *t*‐test was used as indicated. Four outliers (1 from dataset used in Fig 1C, at 12 h, and 3 from dataset in Fig 6F, one from *w* ^*m4h*^, Elba2^−/+^, Elba3^−/+^ each) was removed using the ROUT method (Q = 0.1%). For all qPCR measurements and fluorescence quantification, we used the unpaired one‐ or two‐tailed student *t*‐tests or Mann–Whitney test depending on the normal distribution as measured with the D'Agostino–Pearson normality test or Shapiro–Wilk normality test. As indicated, we used either rpm (Dataset [Link], [Link]) or variance stabilizing transformation (vst) from DEseq2 (version 1.24.00) for normalization of sncRNA and long RNA‐sequencing results. For statistical analysis of sncRNA and long RNA‐seq data, the DEseq2's build‐in Wald test after negative binominal fitting was used. The unpaired one‐ or two‐tailed *t*‐test or Mann–Whitney test was used to test expression rates of specific targets where indicated.

## Author contributions

**Lovisa Örkenby:** Conceptualization; data curation; software; formal analysis; validation; investigation; visualization; methodology; writing – original draft; project administration; writing – review and editing. **Signe Skog:** Software; investigation. **Helen Ekman:** Investigation. **Alessandro Gozzo:** Investigation. **Unn Kugelberg:** Investigation; methodology. **Rashmi Ramesh:** Investigation. **Srivathsa Magadi:** Investigation. **Gianluca Zambanini:** Investigation. **Anna Nordin:** Investigation. **Claudio Cantù:** Resources. **Daniel Nätt:** Conceptualization; data curation; software; formal analysis; methodology; writing – original draft. **Anita Öst:** Conceptualization; resources; supervision; funding acquisition; visualization; methodology; writing – original draft; project administration; writing – review and editing.

## Disclosure and competing interests statement

The authors declare that they have no conflict of interest.

## Supporting information



Appendix

Expanded View Figures PDF

Dataset EV1

Dataset EV2

Dataset EV3

Dataset EV4

Dataset EV5

Dataset EV6

Dataset EV7

Dataset EV8

Dataset EV9

PDF+

## Data Availability

The datasets produced in this study are available in the following databases: sncRNA/long RNA‐seq data: fastq Sequence Read Archive (SRA) BioProject: PRJNA729249 (https://www.ncbi.nlm.nih.gov/bioproject/PRJNA729249) CUT&RUN data: fastq and BAM‐files Sequence Read Archive (SRA) BioProject: PRJNA729249 (https://www.ncbi.nlm.nih.gov/bioproject/PRJNA729249).
