# Supplementary material for: Stress‐sensitive dynamics of miRNAs and Elba1 in Drosophila embryogenesis
Source: Mol Syst Biol. 2023 Mar 20;19(5):e11148. doi: 10.15252/msb.202211148 (PMC10167479; doi:10.15252/msb.202211148)
Supplement: Supplementary file 1 — Appendix [file MSB-19-e11148-s001.pdf]

**Table of Contents**

Page no:

2. **Appendix Figure S1:** The most sensitive period for stress-induced epigenetic program is found in early embryogenesis.

3. **Appendix Figure S2:** Volcano plots of differentially expressed sncRNA reads from stage to stage.

4. **Appendix Figure S3:** Heatmap over unique miRNA reads per embryo stage.

6. **Appendix Figure S4:** GO-term analysis of up- and downregulated genes after heat shock

7. **Appendix Figure S5:** Strong negative correlation between upregulated miRNA and *Elba1*

8. **Appendix Figure S6:** Full western blot images of Ago1 IP experiment on $w^{1118}$ *Drosophila* embryos

9. **Appendix Figure S7:** Overlap of heat shock-induced genes and genes associated to insulator binding factor binding sites.

## Appendix_figure_S1

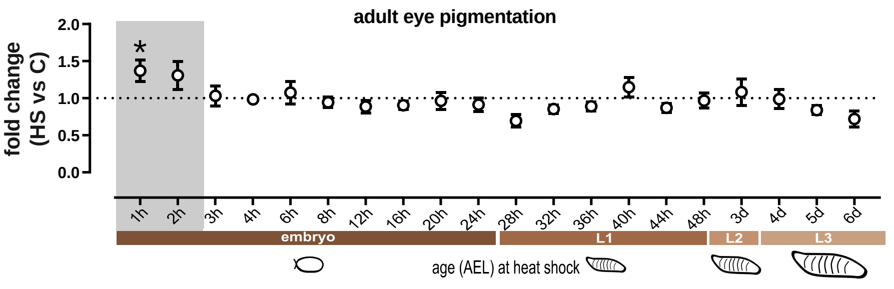


**Appendix Figure S1.** The most sensitive period for stress-induced epigenetic program is found in early embryogenesis.

Spectrophotometric measurement of the heterochromatin eye pigment reporter *white* in 5 day old adult male *Drosophila* $w^{m4h}$ after one session of 1 h heat shock at 37° C performed during different embryonic and larvae stages of development. The starting time after egg laying (AEL) is indicated in the graph. Graph represents the eye pigmentation in relation to the average optical density (OD) of controls (not exposed to heat shock). Heads were measured in groups of 3-10 and normalized to the average OD per head. Presented with ± SEM, * = p = 0.0428 with ordinary one-way ANOVA with Dunnett’s multiple comparison test. Batches measured (same order as in graph) = 9, 9, 12, 12, 14, 10, 8, 11, 8, 7, 7, 5, 3, 8, 8, 6, 5, 5, 6, 5. 2 outliers were removed (one batch from 12h and one from 40 h) using the ROUT method (Q = 0.1%).

**Örkenby_Appendix_figure_S2**

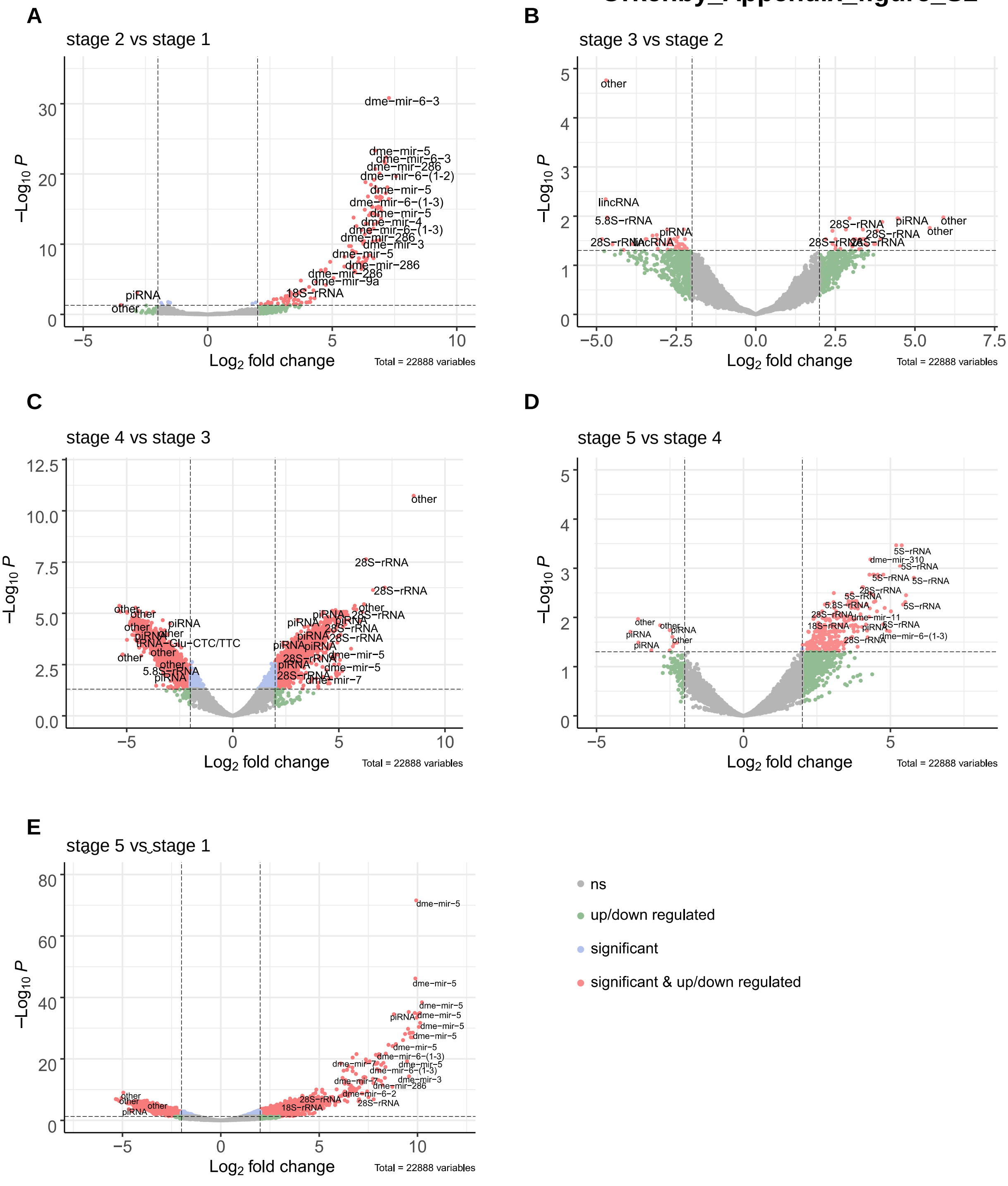


**Appendix Figure S2.** Volcano plots of differentially expressed sncRNA reads from stage to stage.

The volcano plots shows differentially expressed unique sncRNA reads between indicated stages. Red indicates significance at $p \leq 0.05$ (FDR corrected $p \leq 0.05$ using DEseq2's build-in Wald test after negative binominal fitting) and a log2 fold change ≥ or ≤ ± 1.5. (A) Stage 2 vs stage 1 shows that there is an upregulation of the mir-309 cluster already between these stages. (B) Stage 3 vs stage 2, (C) Stage 4 vs stage 3. (D) Stage 5 vs stage 4. (E) Stage 5 vs stage 1. Groups of 5 (stages 1-3) or 4 (stages 4-5) single embryos were sequenced per stage.

Örkenby_Appendix_figure_S3

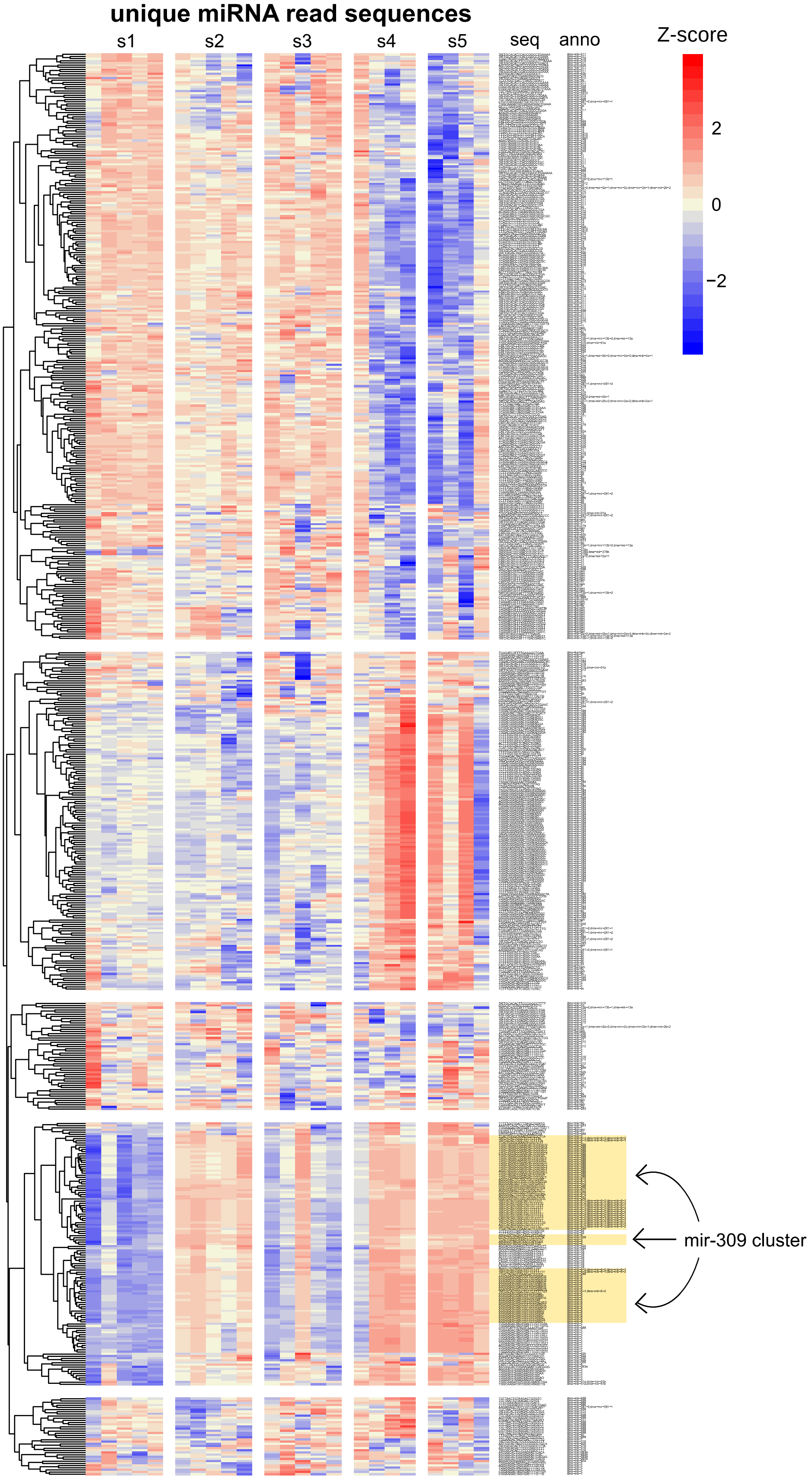

**Appendix Figure S3.** Heatmap over unique miRNA reads per embryo stage. Heatmap of all unique sequences aligning to miRNA per indicated stage. Columns represents unique samples (n = 5 (stages 1-3), 4 (stages 4-5) single embryos per stage). We detected a cluster of miRNA, including the mir-309 cluster (highlighted), that was increased between stage 1 and 2. Color bar represents Z-scores based on rpm expression per miRNA sequence.

A

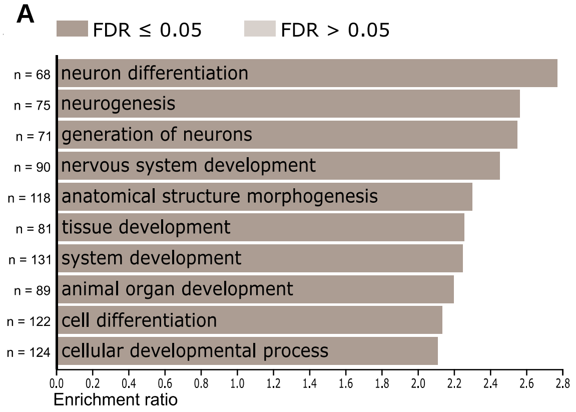


B

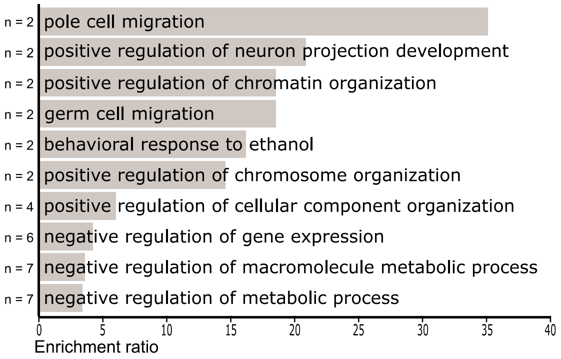


C

| gene name | gene id | molecular function | biological process | cellular component |
|---|---|---|---|---|
| CG1275 | FBgn0035321 | metal ion binding | unknown | late endosome and lysosomal membrane |
| Got1 | FBgn0001124 | L-aspartate:2-oxoglutarate aminotransferase activity | glutamate biosynthetic process | cytosol |
| CG5880 | FBgn0039489 | palmitoyltransferase activity | protein palmitoylation | golgi apparatus |
| Z600 | FBgn0004052 | cyclin binding, protein binding | negative regulation of mitotic nuclear division, development | cytoplasm |
| CG14317 | FBgn0038566 | unknown | protein metabolism | SCF ubiquitin ligase complex |
| term | FBgn0003683 | Developmental protein, DNA-binding | unknown | unknown |
| CG7271 | FBgn0036791 | unknown | unknown | unknown |
| tsg | FBgn0003865 | BMP and heparin binding | response to stimulus, signaling, protein metabolism, development | extracellular region |
| CG6885 | FBgn0036810 | unknown | unknown | unknown |
| dunk | FBgn0083973 | unknown | syncytial embryo cellularization, development | apical cortex, cell periphery, membrane |
| Bro | FBgn0013755 | transcription coactivator activity, DNA binding | regulation of hemocyte proliferation | nucleus |
| Elba1 | FBgn0000227 | chromatin insulator sequence binding, sequence-specific DNA binding | heterochromatin boundary formation, development | chromatin silencing complex, nucleus |
| CG43659 | FBgn0263707 | unknown | unknown | unknown |

**Appendix Figure S4.** GO-term analysis of up- and downregulated genes after heat shock.
GO-term enrichment analysis of all significant genes (FDR corrected $p \leq 0.05$) with log2 fold change ≥ 1 (A) or ≤ -1 (B) after exposure to heat shock during the sensitive period. (A) Upregulated genes (n = 536) were enriched for developmental processes. (B) Downregulated genes (n=42) showed no enrichment. The data was analyzed using WebGestalt (Wang et al.,2017). Top 10 hits from over-representation analysis (ORA) are presented per cluster. (C) Gene ontology annotations of heat shock-reduced genes mapping to cluster 1 (Figure 5C). The GO annotations were taken from Flybase.

# Örkenby_Appendix_figure_S5

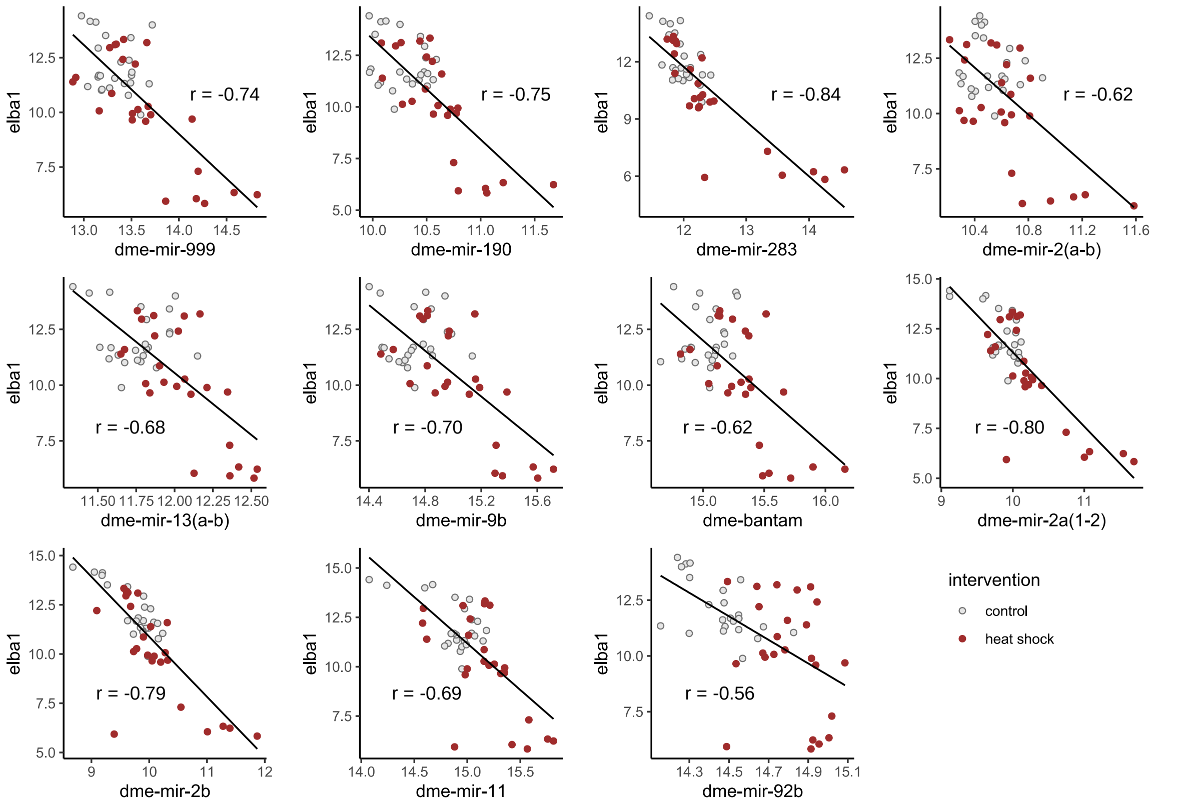


**Appendix Figure S5.** Strong negative correlation between upregulated miRNA and *Elba1*

Correlation between Elba1 and upregulated miRNA based on vst normalized counts. Red circles are heat-shocked single embryos (n = 24), grey circles are control single embryos (n = 24). Pearson's r is associated with each graph. All p-values are < 0.0001.

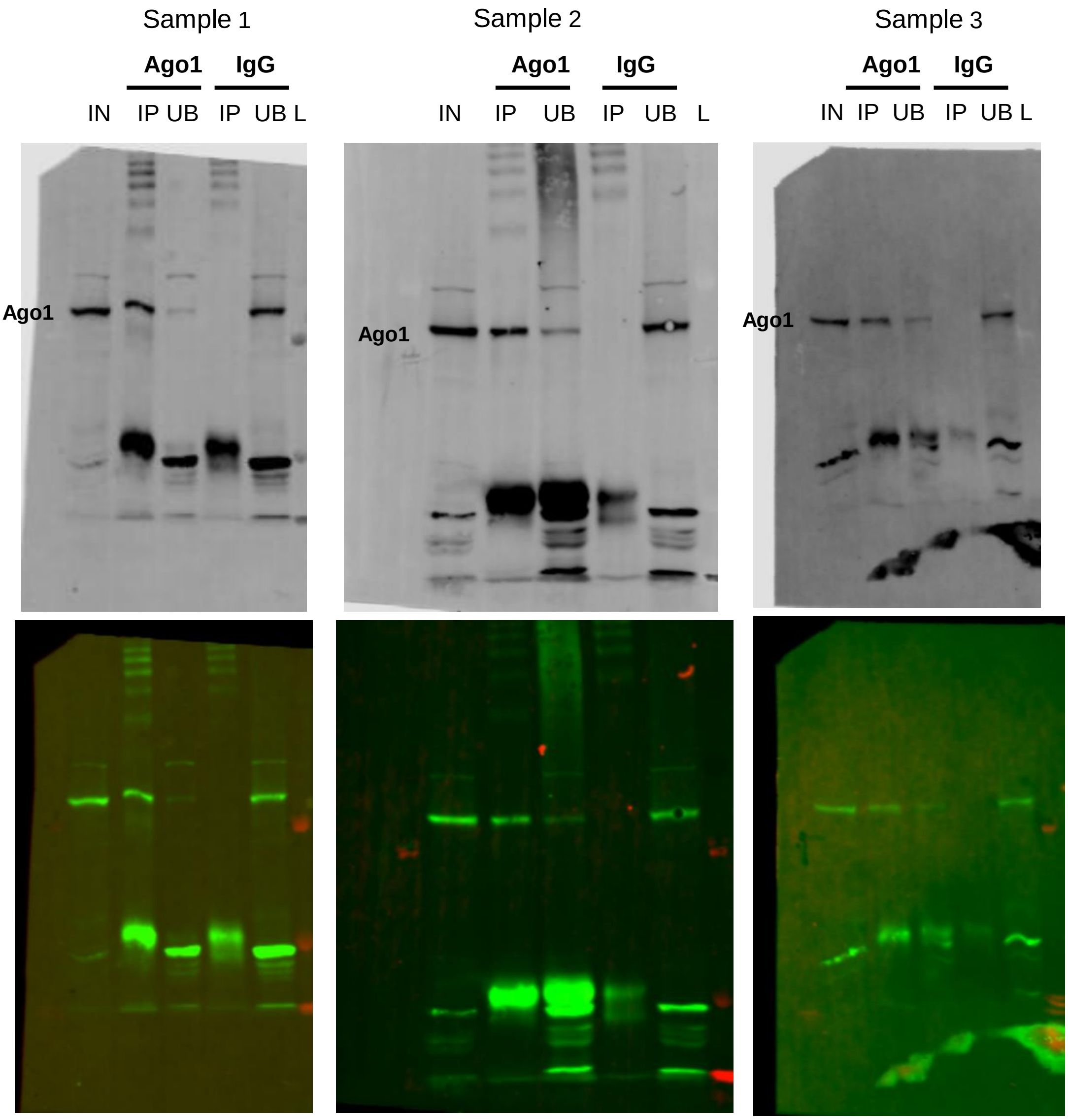


**Appendix Figure S6.** Full western blot images of Ago1 IP experiment on *w*$^{1118}$ *Drosophila* embryos. (black and white) Ago1 band is marked. (color) Ago1 is seen in green channel and the ladder in red. Red band under Ago1 is 100 kDa. There were a total of 3 samples. IN = input, IP = immunoprecipitation, UB = unbound, L = ladder.

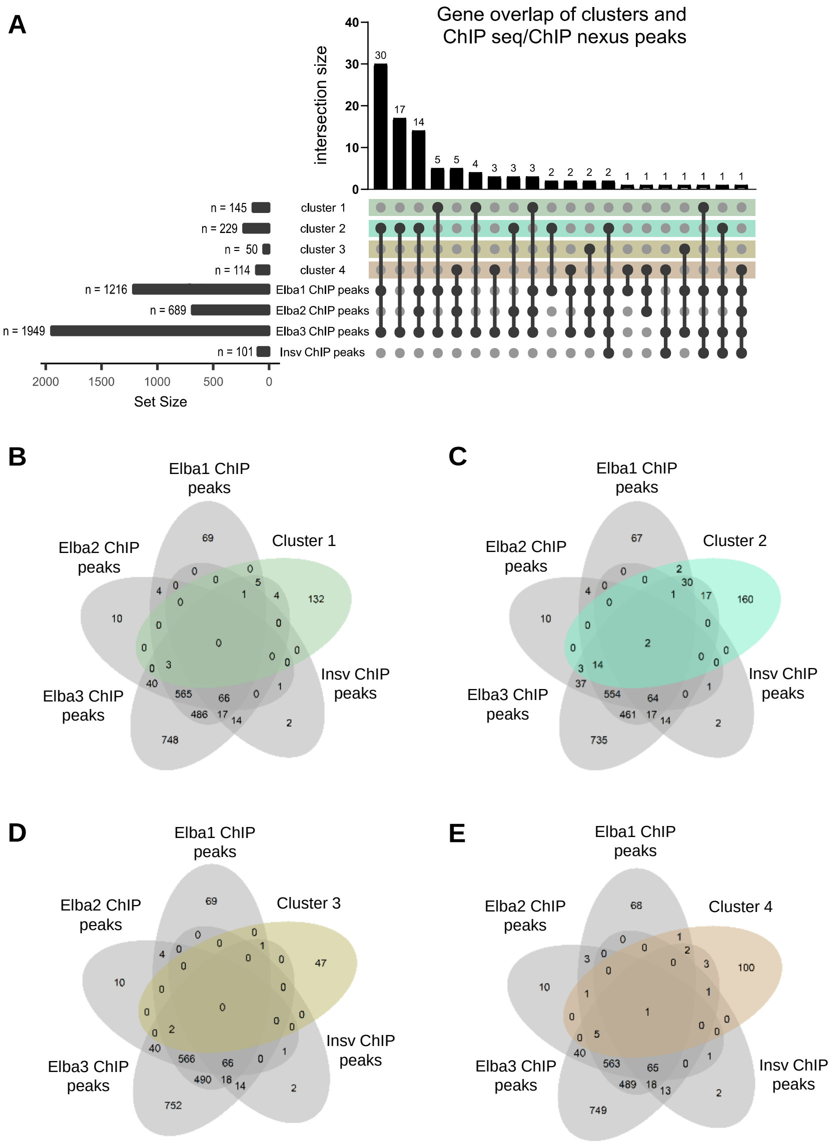


**Appendix Figure S7.** Overlap of heat shock-induced genes and genes associated to insulator binding factor binding-sites.
Venn diagrams showing overlaps between gene clusters identified in Figure 6A and genes associated with published ChIP seq/ChIP nexus peaks (Ueberschär et al., 2019) of the Elba family and Insv insulator binding factors. (A) Intersections between clusters and Elba 1-3 and Insv. (B) Cluster 1, (C) cluster 2, (D) Cluster 3 and (E) cluster 4. Cluster 2 shows more overlap with genes associated to binding sites for the Elba family than the other clusters.
