# Supplementary material for: Stress‐sensitive dynamics of miRNAs and Elba1 in Drosophila embryogenesis
Source: Mol Syst Biol. 2023 Mar 20;19(5):e11148. doi: 10.15252/msb.202211148 (PMC10167479; doi:10.15252/msb.202211148)
Supplement: Supplementary file 2 — Expanded View Figures PDF [file MSB-19-e11148-s009.pdf]

Expanded View Figures

Figure EV1. A heat shock does not induce a temporal bias in the embryonic transcriptome.

A *Drosophila* w^{m4h} embryos were collected in short intervals (30 min) and immediately heat-shocked for 30 min at 37°C (or kept as controls). After being manually staged under a microscope, RNA from the same embryos was used for sequencing of both sncRNA and rRNA-depleted long RNA.

B, C Transcriptome from 4 chronological parts of the nuclear cycle (nc) 14 by Lott *et al* (2011) were subjected to linear regression. (C) Genes were defined as expressed early (slope $\geq$ 1), late (slope ≤ -1), or stable (slope between -1 and 1) Results are mean $\pm$ SEM RPKM.

D (top) Transcriptome of heat-shocked and control embryos from (A) were separated into early, late, and stable genes defined in (C). (bottom) Bar graph of mean rpm of early, late, or stable genes from control and heat-shocked embryos. The nonsignificant (ns) result is based on the unpaired, two-tailed *t*-test. Results are mean $\pm$ SD of $n = 24$ embryos per condition.

E Maternally provided genes as classified in Lott *et al* (2011) showed an equal distribution of up- ($n = 2{,}133$), respectively, downregulated ($n = 1{,}893$) genes between heat-shocked and control embryos. Results are Log2 fold change (heat shock vs. control) of $n = 24$ embryos per condition.

F Early zygotic genes (1-2 h) as classified in De Renzis *et al* (2007) showed an equal distribution of up ($n = 21$), respectively, downregulated ($n = 22$) genes. Results are Log2 fold change (heat shock vs. control) of $n = 24$ per heat shock or control condition.

© 2023 The Authors

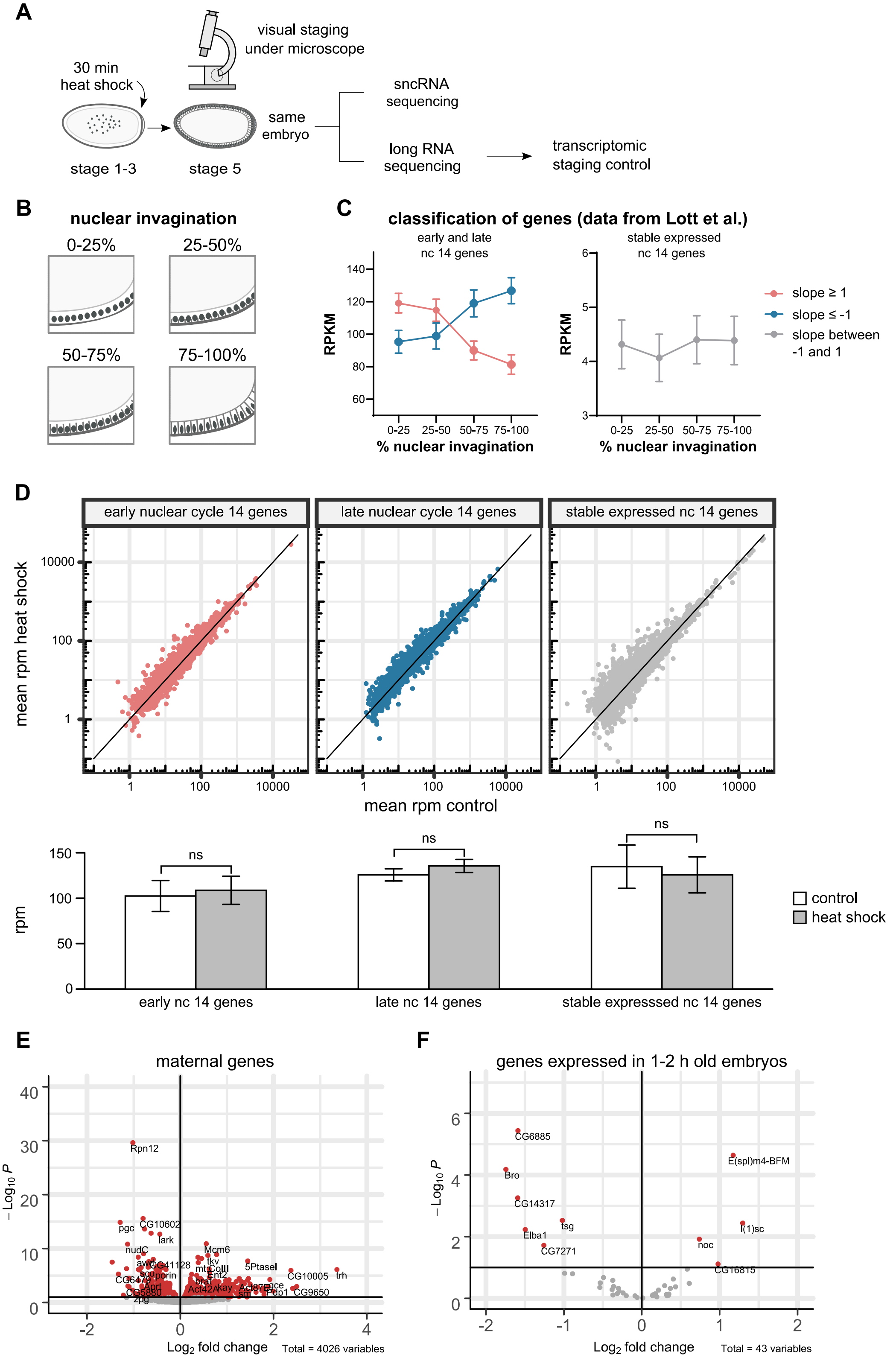
A
30 min heat shock
stage 1-3
stage 5
visual staging under microscope
same embryo
sncRNA sequencing
long RNA sequencing
transcriptomic staging control
B
nuclear invagination
0-25%
25-50%
50-75%
75-100%
C
classification of genes (data from Lott et al.)
early and late nc 14 genes
stable expressed nc 14 genes
RPKM
% nuclear invagination
slope ≥ 1
slope ≤ -1
slope between -1 and 1
D
early nuclear cycle 14 genes
late nuclear cycle 14 genes
stable expressed nc 14 genes
mean rpm heat shock
mean rpm control
rpm
ns
control
heat shock
early nc 14 genes
late nc 14 genes
stable expresssed nc 14 genes
E
maternal genes
F
genes expressed in 1-2 h old embryos
Log2 fold change
−Log10 P
Total = 4026 variables
Total = 43 variables
Rpn12
CG6885
Bro
CG14317
tsg
Elba1
CG7271
E(spl)m4-BFM
l(1)sc
noc
CG16815

Figure EV1.

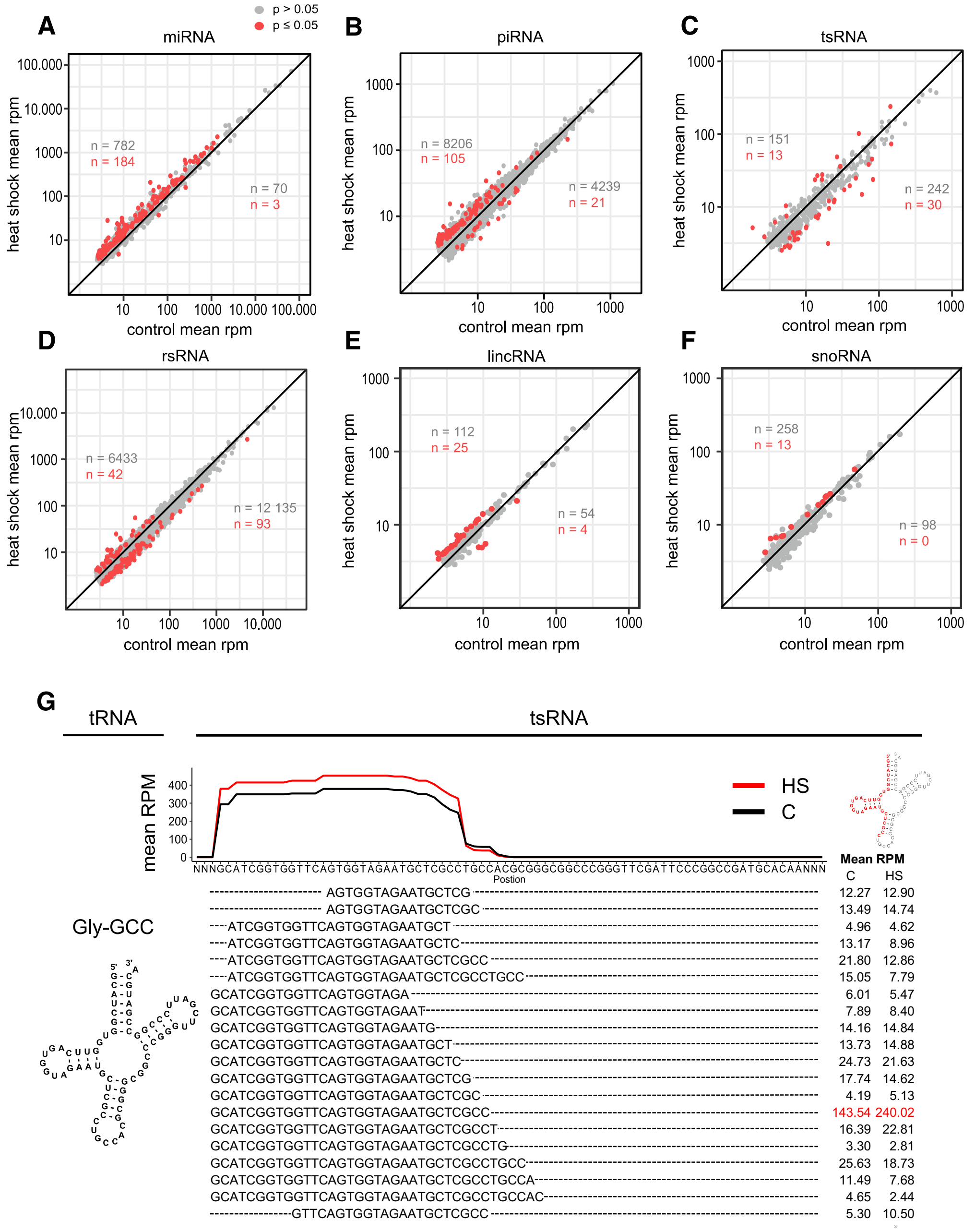

Figure EV2. Unique sncRNA sequences after exposure to heat shock during the sensitive period.

A–F Scatter plots comparing rpm normalized expression levels of unique miRNAs (A), piRNAs (B), tRNA- (C) rRNA fragments (D), lincRNAs (E), snoRNAs (F) between control and heat-shocked embryos. Red = FDR corrected $P \leq 0.05$, gray = FDR corrected $P > 0.05$ using the DEseq2's build-in Wald test after negative binominal fitting.

G Line graph shows mean rpm coverage of reads mapping to tRNA-Gly-GCC. Mean rpm of each unique sequence contributing to the line plots is presented below the graph. The most significantly differentially expressed sequence is marked in red.

Data information: FDR corrected $P \leq 0.05$ using the DEseq2's build-in Wald test after negative binominal fitting. C, control sample; HS, heat-shocked. $n = 24$ single w^{m4h} embryos per condition.

 © 2023 The Authors

Figure EV3. CUT&RUN tracks of Elba1-GFP in *Drosophila* embryo at representative loci.

CUT&RUN was made using a GFP antibody on Elba1-GFP or w^{1118} stage 5 embryos. The Elba1-GFP embryos were either exposed to 30 min heat shock pre-MBT, or kept as controls.

A Heatmap and profiles of peak scores centered over TSS at genes from clusters 1–4 (Fig 6A). Peak scores are based on 5 merged samples of 20 embryos each per condition.

B Quantification of mean gene peak score per indicated gene cluster (Fig 6A) at 250 bp upstream TSS to TSS. Cluster 2 shows increased Elba1 binding at the TSS compared with other gene clusters, and this binding is higher in control than in heat-shocked embryos. Results are mean ± SEM, *$P = 0.0314$, **$P = 0.0022$, ****$P < 0.0001$ using two-way ANOVA with the Šídák's multiple comparisons test. Peak scores are based on 5 merged samples of 20 embryos each per condition.

C, D CUT&RUN tracks showing two representative loci on chromosome 3 together with heat shock-induced genes from cluster 2 in Fig 6A.

E CUT&RUN peaks showing the Fab-7 region of the bithorax complex, which is a well-known Elba-binding site. The Elba binding motifs are marked in red. All tracks are merged per condition $n = 5$ (controls and heat shock), $n = 2$ w^{1118}.

© 2023 The Authors

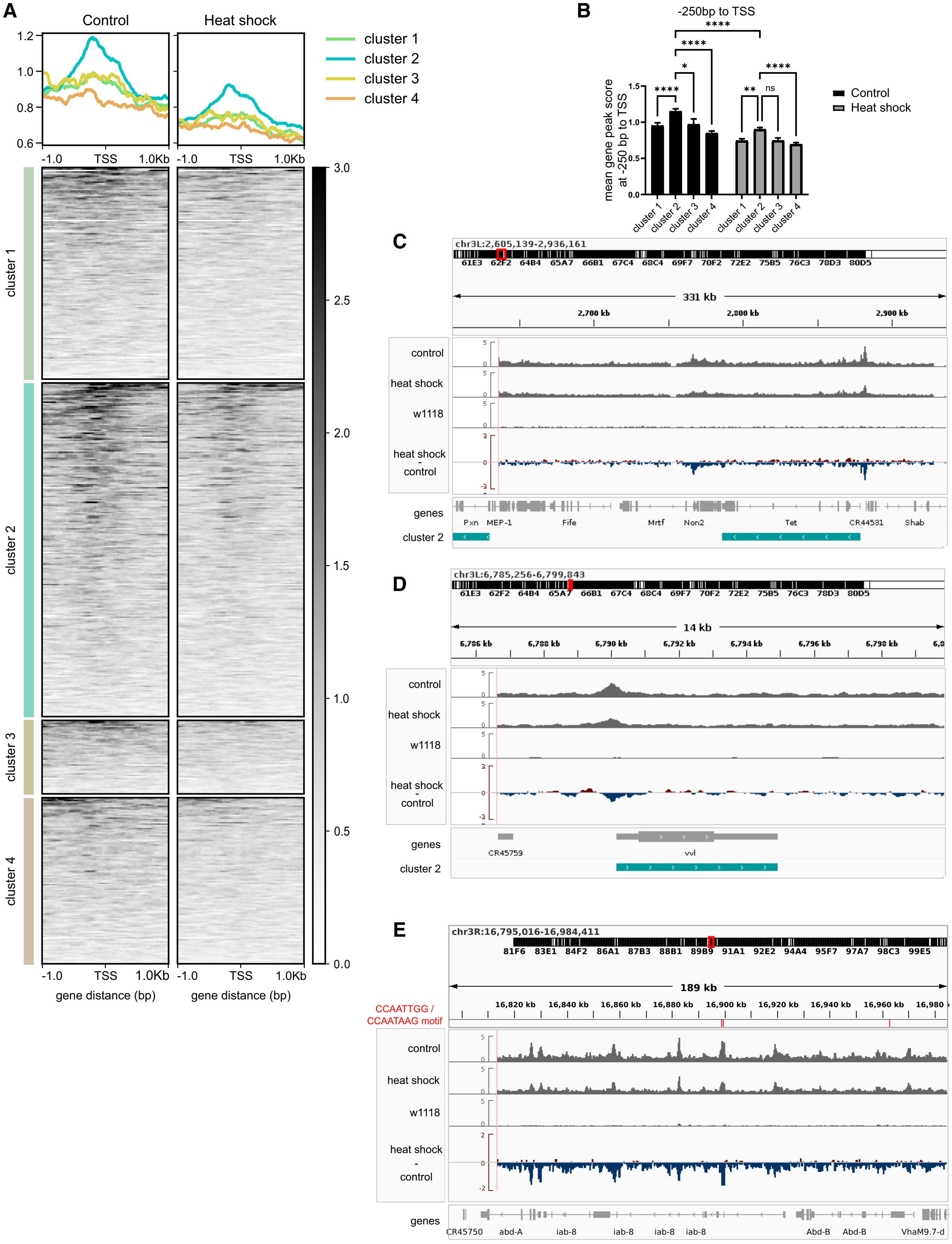

Figure EV3.

© 2023 The Authors

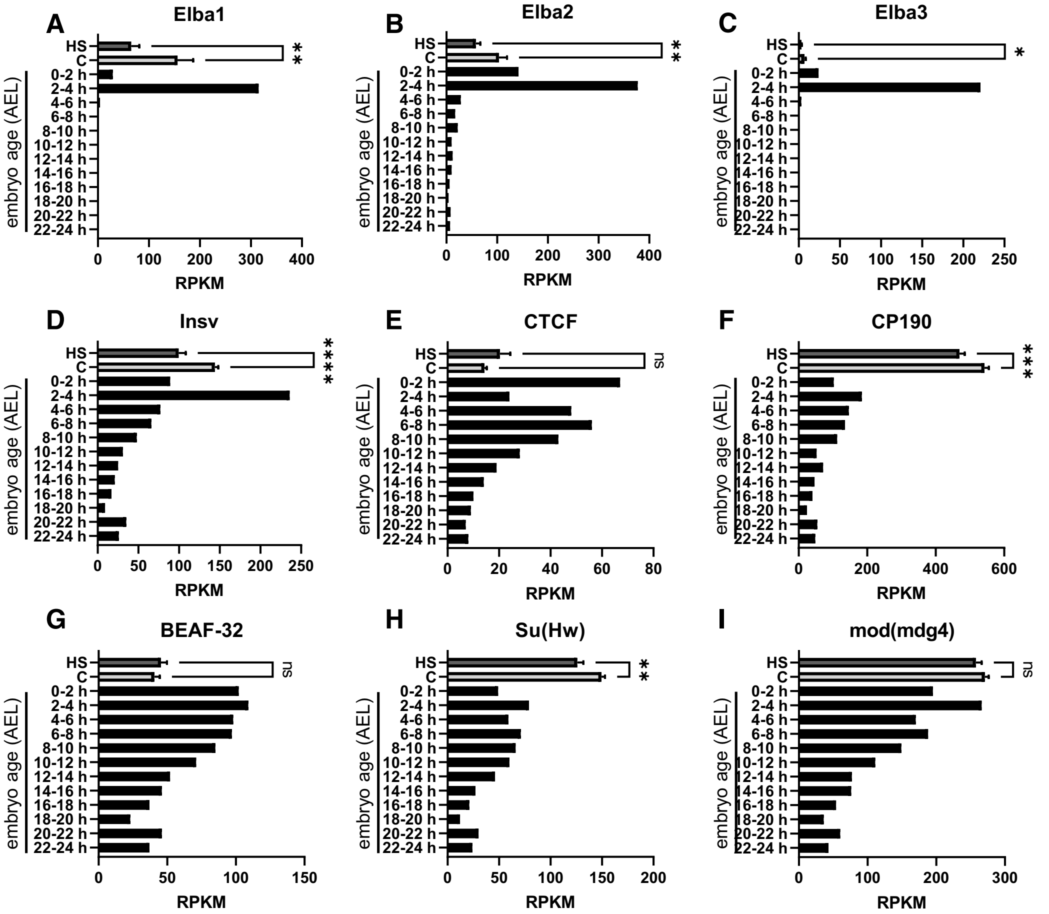

Figure EV4. Embryonic expression of insulator-binding factors.

Expression levels of known insulator-binding factors at different embryonic ages and in control or heat-shocked stage 5 embryos. Data were extracted from modENCODE, AEL = after egg laying. C = control and HS = heat-shocked w^{m4h} stage 5 embryos. ns = nonsignificant. n = 24 single embryos per condition.

A–C Elba factors are specifically expressed (especially Elba1 and Elba3) during pre-MBT and MBT. Error bars represents mean ± SEM, Elba1 P (**) = 0.0014, Elba2 P (**) = 0.0094, Elba3 P (*) = 0.0128, using an unpaired two-tailed Mann–Whitney test.

D Insv is highly expressed in 2- to 4-h-old embryos but continue to be expressed throughout embryogenesis. Error bars represent mean ± SEM, P (****) ≤ 0.0001 using an unpaired two-tailed t-test.

E–I The other factors are also expressed throughout embryonic development but with a declining trend. Error bars represents mean ± SEM, CP190 P (***) = 0.0009, Su(Hw) P (**) = 0.0017 using an unpaired two-tailed t-test.

© 2023 The Authors
