# Supplementary material for: Stress‐sensitive dynamics of miRNAs and Elba1 in Drosophila embryogenesis
Source: Mol Syst Biol. 2023 Mar 20;19(5):e11148. doi: 10.15252/msb.202211148 (PMC10167479; doi:10.15252/msb.202211148)
Supplement: Supplementary file 13 [file MSB-19-e11148-s011.pdf]

Stress-sensitive dynamics of miRNAs and Elba1 in Drosophila embryogenesis

Lovisa Örkenby, Signe Skog, Helen Ekman, Alessandro Gozzo, Unn Kugelberg, Rashmi Ramesh, Srivathsa Magadi, Gianluca Zambanini, Anna Nordin, Claudio Cantù, Daniel Nätt, and Anita Öst
DOI: 10.15252/msb.202211148

Corresponding author(s): Anita Öst (anita.ost@liu.se)

Review Timeline:

Submission Date:	29th May 22
Editorial Decision:	27th Jun 22
Revision Received:	26th Nov 22
Editorial Decision:	17th Jan 23
Revision Received:	15th Feb 23
Accepted:	23rd Feb 23

Editor: Maria Polychronidou

Transaction Report:

(Note: With the exception of the correction of typographical or spelling errors that could be a source of ambiguity, letters and reports are not edited. Depending on transfer agreements, referee reports obtained elsewhere may or may not be included in this compilation. Referee reports are anonymous unless the Referee chooses to sign their reports.)

Thank you again for submitting your work to Molecular Systems Biology. We have now heard back from the two referees who agreed to evaluate your study. As you will see below, the reviewers raise a series of concerns, which preclude the publication of your study in its current form. Overall, the reviewers think that as it stands the study seems rather preliminary and the main conclusions are not convincingly supported.

However, given that the reviewers acknowledge that the presented findings seem relevant for the field, we have decided to offer you the chance to address the issues raised in a major revision. Without repeating all the points listed below, some of the more fundamental issues are the following:

- The causal role of Elba in heat shock-induced gene expression should be better supported by additional experiments.

- Further experiments should be performed to demonstrate that heat shock leads to physiologically relevant stabilization of miRNAs that target the elba1 mRNA. Moreover, analyses explaining how miRNAs target the elba1 mRNA should be included.

- Additional analyses and controls are needed to better support the reported effects of heat shock on miRNA levels.

- The reviewers also list several technical concerns related to the data analysis and interpretation, which need to be convincingly addressed.

All issues raised by the reviewers need to be satisfactorily addressed as they are related to the conclusiveness of the main findings. Both reviewers make constructive suggestions on how to improve the study. As you may already know, our editorial policy allows in principle a single round of major revision. It is therefore essential to provide responses to the reviewers' comments that are as complete as possible. Given that the required revisions are quite substantive, we would understand if you would choose to not perform all these additional experiments and would decide to submit the study elsewhere. If you decide to revise and re-submit to MSB and would like to discuss your revision plan with me please feel free to get in touch.

Reviewer #1:

Örkenby et al present evidence that early embryonic stress in Drosophila can indirectly affect adult heterochromatin, which they propose arises from inhibition of wild-type degradation of maternal miRNAs, which in turn aberrantly downregulate the zygotic chromatin factor Elba1. This is an intriguing and exciting possibility, and while the authors present multiple pieces of data in support of their model, I think the evidence falls just short for the authors to make their claims as strongly as they do.

I think the authors would need to demonstrate that heat shock leads to physiologically relevant stabilization of miRNA that target elba1 mRNA. This could be accomplished using a 3'UTR reporter for elba1 mRNA and measuring accelerated decay upon heat shock; but there may be other options as well. Surprisingly, the authors do not discuss how elba1 mRNA could be targeted: the 3'UTR of the elba1 mRNA is relatively short and does not seem to have target sequences for any of the miRNAs in question, according to TargetScan.

Lacking this piece of data, I think the authors would need to moderate their claims (including the title). There are caveats to their current interpretation:

- The effects of heat shock on miRNA levels seem overall mild, with most miRNAs up by less than 2-fold
- It is challenging to claim that any miRNA levels are reduced during the MZT, because, as the authors note, accurate quantification and comparison between stages is affected by the very large changes to the overall small RNA repertoire. An external reference (e.g., spike-in) could have helped.
- Dcr-1 mutants likely have differences in their maternal contribution beyond just miRNAs, which could significantly affect gene regulation during the MZT

Additional comments:
- The authors observe that short cell cycles in the early embryo a) account for delayed ZGA and the need for maternal factors (Intro); and b) restrict early transcription to include only small genes like miRNAs (Discussion). While true to some extent, Syed et al (PNAS 2021) demonstrate that the nuclear-cytoplasmic ratio does influence ZGA timing for at least some fly genes. Moreover, miRNAs are often transcribed as larger primary transcripts that are not necessarily shorter than other mRNA (it is of course possible that aborted transcripts from a miRNA cluster could yield functional mature miRNA, but that's a somewhat different claim than what the authors are saying)
- Using a t test for sequencing data is not really appropriate (Fig 3d, 4f-h). A non-parametric test (Wilcoxon rank sum) would probably be fine here, given how many replicates there are, but in general one should avoid these sorts of bulk quantifications and instead rely on statistical tests (e.g., DESeq2) on individual RNA species to together demonstrate systematic differences

Reviewer #2:

In this manuscript, Örkenby and colleagues successfully recapitulate the suppression of variegation obtained in the adult eye of wm4h Drosophila melanogaster, upon heat shock applied during the early stages of embryogenesis.
Here, the authors studied the impact of the heat shock on small ncRNAs, after having characterized their expression in untreated wildtype Drosophila embryos from stage 1 to stage 5. Upregulation of several miRNA members (both maternally provided or zygotically transcribed) upon heat shock treatment represents the most compelling finding. Next, the authors find Elba1 among the significantly downregulated genes upon heat shock treatment. They propose that Elba1 downregulation is due to the increased expression of some specific miRNAs caused by heat shock treatment. Moreover, the consequent Elba1 downregulation is responsible for the suppression of variegation in the adult eye because of impaired transcriptional repression of some specific genes.

This work shows interesting findings as a detailed characterization of small RNAs during the first stages of Drosophila embryogenesis and the impact of heat shock treatment on their expression and on the mRNAs during stage 5. We suggest a revision of the manuscript with some additional experiments as detailed below.

MAJOR POINTS:

The authors claim that the global increase in the vast majority of miRNAs species upon heat shock is due to both reduced degradation levels in maternally deposited miRNAs and in increased transcription of zygotically expressed pre-miRNA genes. Their conclusion is supported by their categorization of maternal vs. zygotic miRNA, based on the small RNA-sequencing performed in wild-type embryos.

1) Although reduction or complete loss of degradation of maternally deposited miRNAs upon heat shock is highly plausible, the authors should couple small RNA-seq in control and in heat-shocked embryos with the maternal depletion of Wispy or other factors that could mediate miRNA degradation. In this context, the amount of miRNAs should not be different in control KD, heat-shocked KD and heat-shocked WT embryos.

2) In order to prove that the Elba complex is responsible for the correct expression of HS-induced genes, as inferred in the manuscript, the authors should perform ChIP-seq for the Elba complex component also in HS-treated embryos. Correlation between Elba peaks in wildtype and upregulation of genes associated with these peaks in HS-treated embryos is not sufficient to prove causality.

3) In figure 2, the authors speculate that the increase of mir-309 cluster members in the very first stages is due to an early activity of Zelda. Zelda ChIPs are publicly available and the authors should check if the mir-309 cluster is bound by Zelda in the early embryo.

4) How did the authors normalize for variation in the total amount of small RNAs? Have the authors assumed that total amounts were constant for each stage of embryogenesis or upon heat shock, and if yes how this assumption is justified?

MINOR POINTS

Introduction:
- Drosophila embryogenesis lasts 22 hours, not 24 hours
- The authors wrote: "it is easy to envision a general role for sncRNA, including miRNA, in initiating or influencing the early higher-order chromatin landscape". How miRNA could do that if not indirectly? It is possible that the authors hypothesized a change in rasiRNAs or in piRNAs and they could instead find a change in miRNAs upon heat shock. Nonetheless, a change in miRNA population cannot directly affect higher-order chromatin landscape as stated in the text. Therefore, the authors should remove "including miRNA".

Figure 1:
- The authors wrote: "we found that the first two hours in embryogenesis are the most sensitive period for heat shock induction of long-term effects on heterochromatin". Maybe it would be more correct to claim that it is the only period? Indeed, heat shock performed by the authors in any other stage does not significantly affect eye pigmentation according to their results.

- Figure 1B: 27h at 22C?

Figure 2:
- The authors wrote: "The first two hours of Drosophila embryogenesis, entailing stages 1-3 (at 22{degree sign} C)". Stage 3 lasts till 1h 20min AEL. From 1h 20min to 2h 10min AEL there is stage 4. The authors should change it with 1-4.

- Figure 2A: the authors should link embryonic stages with nuclear cycles inside the panel. Cycle 14 starts at 2h 10min AEL, therefore most of stage 4 (from cycle 10 to cycle 13) lays inside the first 2 hours of embryogenesis.

- Figure 2B and figure 2C could be merged, by simply add percentages to panel 2C for each RNA class. One extra panel could be added to show how many miRNAs, piRNAs tsRNAs, etc. are individually up or downregulated in each embryonic stage as unique sequences, instead of showing the total amount that could be biased by few overrepresented members.

Figure 3:
- From panel B to panel F the authors demonstrate the validity of their system and a lack of any temporal bias among groups. All this data can be moved to supplementary figures. Panel 3A could be included in Figure 4

- Figure 3C: why the 2 y-axes are so much different in their RPKM ranges?

Figure 4:
- Figure 4B is redundant with 4A. It could be removed

- Figure 4C: together with miRNAs, all the other classes combined (all small RNAs minus miRNAs) should be shown in control and upon HS. Their lack of global difference would be a good comparison with the miRNAs, which are instead affected.

- Figure 4D: this panel is misleading as it shows only the RNAs that are significantly up or downregulated upon HS. It would be much better to show the whole populations as violin plots, together with the number of detected and significantly up or downregulated members per each RNA class. Indeed, the bimodal distributions observed in tsRNA and rsRNA simply represent the tails of of normally distributed samples. Furthermore, snoRNAs are mostly not affected as shown in supplementary figure 4. The 13 (modestly) upregulated snoRNAs out of 369 are clearly not representative of the whole population. Although specified in figure 4D, its representation is highly misleading for the reader. From supplementary figure 4, it is pretty clear that the only relevant effects of HS treatment are 1) global upregulation of several miRNA species; 2) up or downregulation of specific tsRNA species. These findings should be highlighted in the text and any other mention to snoRNAs or piRNAs should be removed, unless to claim for a lack of any significant effect on their populations upon HS treatment.

- Figure 4G: why only 3 zygotic miRNAs are shown in contrast to the several maternal miRNAs shown? Are those the only zygotic ones to be affected upon HS treatment?

- Supplementary 4H and 4I should be integrated in the main figure

- If transcription of zygotic miRNA is affected by heat shock treatment, how do the authors explain that zelda-dependent mir-309 cluster is not affected?

Figure 5:
- Figure 5B: Among all the 459 downregulated genes upon HS treatment, the authors should check for any overlap with a list of all the known suppression of variegation genes, as they did for the few genes shown in 5B. Similarly, the authors should compare the list of the 1134 upregulated genes with enhancer of variegation genes. Finding that other genes involved in the variegation are also affected by the heat shock could help to explain the additional contributions not linked to Elba1 function.

- Figure 5J: the authors should show a representative picture of Elba1-GFP embryos upon heat shock treatment

Response to Reviewer #1:

Thank you for the critique. In response the two reviews we have included a number of new experiments that strengthen the manuscript. Please find below a synopsis of the major additions, followed by the point-by-point response to your comments.

We have performed CUT&RUN for Elba1-GFP on stage 5 embryos with or without heat shock (Fig 6C-D, Fig EV3). In short, heat-shocked embryos displayed less Elba1-GFP peaks compared to the control (Fig 6D, Fig EV3). This was most pronounced in genes that we earlier defined as inversely correlated to Elba1 (cluster 2, Fig 6A, C-D). Furthermore, to strengthen the notion that miRNA is regulating mRNA levels of Elba1 we have done a pull-down of the RISC complex via Ago1 and performed qPCR against Elba1. This experiment showed a clear enrichment of Elba1, thus providing direct evidence for the interaction between Elba1 and the RISC complex (Fig 5F). In addition, we have performed an *in silico* analysis of predictive miRNA targets for Elba1 (Fig 5C). This analysis identified two of the candidate miRNAs with a perfect match in the 3´-UTR, miR-13b and miR-2a-2/2c. These two miRNAs are in the cluster of miRNAs that we earlier detected to have the strongest negative correlation to the identified pre-MBT genes (including Elba1)(Fig 4D). Together with our earlier experiment showing that heat shock induced down-regulation of Elba1 is *Dicer-1* dependent (Fig 5D), these data paint a picture where miRNA is important in finetuning the amount of Elba1 in early embryogenesis.

In addition to these experiments, we have shortened the discussion, rewritten the abstract, and added text in the result section to match the new data. Changes in the text is shown with track changes and row numbers are referred to with track changes on. Please note that figure 3-6 all have updates in response to reviewer comments. For example, the figure showing that the staging is correct is moved to the Expanded View (Fig EV1) (Old fig 3).

Örkenby et al present evidence that early embryonic stress in Drosophila can indirectly affect adult heterochromatin, which they propose arises from inhibition of wild-type degradation of maternal miRNAs, which in turn aberrantly downregulate the zygotic chromatin factor Elba1. This is an intriguing and exciting possibility, and while the authors present multiple pieces of data in support of their model, I think the evidence falls just short for the authors to make their claims as strongly as they do.

I think the authors would need to demonstrate that heat shock leads to physiologically relevant stabilization of miRNA that target elba1 mRNA. This could be accomplished using a 3'UTR reporter for elba1 mRNA and measuring accelerated decay upon heat shock; but there may be other options as well.

Thank you for this suggestion. We interpret that the reviewer suggests a 3'UTR luciferase reporter assay in *Drosophila* embryos, since a heat shock in a cell line cannot mimic the situation of retention of maternally loaded miRNA. There are, to our knowledge, no such flies available and we have therefore made an alternative experiment to strengthen the miRNA-Elba1 conclusion.

To directly test if elba1 interacts with the miRNA-RISC complex we have now performed a pull-down with Ago1 followed by qPCR for Elba1. The result shows a clear enrichment of Elba1 in the Ago1 IP fraction (New Fig 5F). Thereby strengthening the notion that Elba1 is targeted by miRNA by its association to Ago1.

Surprisingly, the authors do not discuss how elba1 mRNA could be targeted: the 3'UTR of the elba1 mRNA is relatively short and does not seem to have target sequences for any of the miRNAs in question, according to TargetScan.

Thank you for pointing this out. We have now included an *in-silico* data analysis of potential miRNA target sites on the Elba1 transcript by comparing the Elba1 transcript (Ensembl) and the seed sequences of target miRNA (from TargetScan Fly). The miRNAs were mapped using SnapGene and the result is shown in Fig 5C. The text matching this figure reads (row 296-301):

"To investigate if our candidate miRNAs (Fig 4D) had potential target sites on Elba1, we performed an in-silico analysis comparing miRNA seed sequences complementary to the Elba1 transcript (Fig 5C). We found that two of the candidate miRNAs (mir-13 and mir-2) had a perfect 8 nucleotide seed match, and several had a one-mismatch seed match (mir-190, mir-11, mir-999 and bantam) at the Elba1 3'UTR."

Lacking this piece of data, I think the authors would need to moderate their claims (including the title). There are caveats to their current interpretation:

- The effects of heat shock on miRNA levels seem overall mild, with most miRNAs up by less than 2-fold

Yes, this is true. Most miRNA are up by less that 2-fold. On the other hand, it is on a broad front. As we mainly detect elevated levels of maternal miRNAs, we expect that heat shock keeps them from degradation. We do therefore not expect huge differences in expression.

- It is challenging to claim that any miRNA levels are reduced during the MZT, because, as the authors note, accurate quantification and comparison between stages is affected by the very large changes to the overall small RNA repertoire. An external reference (e.g., spike-in) could have helped.

We agree with the reviewer that it is difficult to claim that there are changes in miRNA during the MTZ. If we would do it again, we would add a spike-in. There is however a published dataset with miRNA in *Drosophila* embryogenesis that has used a spike-in (Zhou et al, 2018). Even though they don't have the same temporal resolution as us, their classification of maternal and zygotic miRNA largely agrees with ours. We have now added the following text in the manuscript to cover this question (row 233-239):

"To confirm that this classification was correct, we compared our results with the miRNA expression profiles of 0-2 and 2-4 h old embryos made by Zhou et al (Zhou et al, 2018), and found that our classifications largely aligns with theirs."

In addition, we have changed the phrasing in the manuscript to *proportions* of different biotypes to make it clear that it is relative values. For example, on rows 137-144:

"As expected during the maternal to zygotic transition, stage 3 is characterized by an increased proportion of degradation products ("other", Fig 2B) which then decrease at the end of MZT. We also noted that there is a larger proportion of rRNA fragments (rsRNAs) in stage 1 than in the other stages, while tRNA fragments (tsRNAs) are present at low levels at all stages (Fig 2B-C). In contrast, piRNAs are highly expressed throughout all stages. The most striking change, however, was the proportion of miRNA. It increased from approximately 9% in stage 1 embryos to over 50 % in stage 5 (Fig 2B). "

- Dcr-1 mutants likely have differences in their maternal contribution beyond just miRNAs, which

could significantly affect gene regulation during the MZT

We choose to do this experiment using a Dicer-1 mutant to have as insignificant effect as possible on other sncRNA species (Lee et al, 2004). As described above, we have now added experimental as well as *in-silico* evidence supporting that Elba1 is targeted by miRNA.

Additional comments:
- The authors observe that short cell cycles in the early embryo a) account for delayed ZGA and the need for maternal factors (Intro); and b) restrict early transcription to include only small genes like miRNAs (Discussion). While true to some extent, Syed et al (PNAS 2021) demonstrate that the nuclear-cytoplasmic ratio does influence ZGA timing for at least some fly genes. Moreover, miRNAs are often transcribed as larger primary transcripts that are not necessarily shorter than other mRNA (it is of course possible that aborted transcripts from a miRNA cluster could yield functional mature miRNA, but that's a somewhat different claim than what the authors are saying)

We agree that this section was a bit speculative and have now deleted that part from the discussion.

- Using a t test for sequencing data is not really appropriate (Fig 3d, 4f-h). A non-parametric test (Wilcoxon rank sum) would probably be fine here, given how many replicates there are, but in general one should avoid these sorts of bulk quantifications and instead rely on statistical tests (e.g., DESeq2) on individual RNA species to together demonstrate systematic differences.

We have now revised the statistics throughout the manuscript and have tested the normal distribution and chosen tests accordingly. E.g. for graphs fig 4f-h (now Fig 3E-G) we changed from t-test to the non-parametric Mann-Whitney test, which changed our conclusion on zygotic miRNA. We are very thankful for the constructive review and think that this input has led to a substantial improvement of the manuscript.

Response to Reviewer #2:

Thank you for the critique. In response the two reviews we have included a number of new experiments that strengthen the manuscript. Please find below a synopsis of the major additions, followed by the point-by-point response to your comments.

We have performed CUT&RUN for Elba1-GFP on stage 5 embryos with or without heat shock (Fig 6C-D, Fig EV3). In short, heat shocked embryos displayed less Elba1-GFP peaks compared to the control (Fig 6D, Fig EV3). This was most pronounced in genes that we earlier defined as inversely correlated to Elba1 (cluster 2, Fig 6A, C-D). Furthermore, to strengthen the notion that miRNA is regulating mRNA levels of Elba1 we have done a pull-down of the RISC complex via Ago1 and performed qPCR against Elba1. This experiment showed a clear enrichment of Elba1, thus providing direct evidence for the interaction between Elba1 and the RISC complex (Fig 5F). In addition, we have performed an *in silico* analysis of predictive miRNA targets for Elba1 (Fig 5C). This analysis identified two of the candidate miRNAs with a perfect match in the 3´-UTR, miR-13b and miR-2a-2/2c. These two miRNAs are in the cluster of miRNAs that we earlier

detected to have the strongest negative correlation to the identified pre-MBT genes (including Elba1)(Fig 4D). Together with our earlier experiment showing that heat shock induced down-regulation of Elba1 is *Dicer-1* dependent (Fig 5D), these data paints a picture where miRNA is important in finetuning the amount of Elba1 in early embryogenesis.

In addition to these experiments, we have shortened the discussion, rewritten the abstract and added text in the result section to match the new data. Changes in the text is shown with track changes and row numbers are referred to with track changes on. Please note that figure 3-6 all have updates in response to reviewer comments. For example, the figure showing that the staging is correct is moved to the Expanded View (Fig EV1) (Old fig 3).

In this manuscript, Örkenby and colleagues successfully recapitulate the suppression of variegation obtained in the adult eye of wm4h Drosophila melanogaster, upon heat shock applied during the early stages of embryogenesis.

Here, the authors studied the impact of the heat shock on small ncRNAs, after having characterized their expression in untreated wildtype Drosophila embryos from stage 1 to stage 5. Upregulation of several miRNA members (both maternally provided or zygotically transcribed) upon heat shock treatment represents the most compelling finding. Next, the authors find Elba1 among the significantly downregulated genes upon heat shock treatment. They propose that Elba1 downregulation is due to the increased expression of some specific miRNAs caused by heat shock treatment. Moreover, the consequent Elba1 downregulation is responsible for the suppression of variegation in the adult eye because of impaired transcriptional repression of some specific genes.

This work shows interesting findings as a detailed characterization of small RNAs during the first stages of Drosophila embryogenesis and the impact of heat shock treatment on their expression and on the mRNAs during stage 5. We suggest a revision of the manuscript with some additional experiments as detailed below.

MAJOR POINTS:

The authors claim that the global increase in the vast majority of miRNAs species upon heat shock is due to both reduced degradation levels in maternally deposited miRNAs and in increased transcription of zygotically expressed pre-miRNA genes. Their conclusion is supported by their categorization of maternal vs. zygotic miRNA, based on the small RNA-sequencing performed in wild-type embryos.

1) Although reduction or complete loss of degradation of maternally deposited miRNAs upon heat shock is highly plausible, the authors should couple small RNA-seq in control and in heat-shocked embryos with the maternal depletion of Wispy or other factors that could mediate miRNA degradation. In this context, the amount of miRNAs should not be different in control KD, heat-shocked KD and heat-shocked WT embryos.

We agree that it would be interesting to investigate the mechanism for heat shock induced retention of maternal miRNA. The only reported mechanism for degradation of maternal miRNA is to our knowledge via Wispy. Unfortunately, maternal Wispy depletion result in early meiotic arrest (Brent et al. 2000) and this experiment is therefore impossible to perform in stage 5 embryos.

2) In order to prove that the Elba complex is responsible for the correct expression of HS-induced genes, as inferred in the manuscript, the authors should perform ChIP-seq for the Elba complex component also in HS-treated embryos. Correlation between Elba peaks in wildtype and upregulation of genes associated with these peaks in HS-treated embryos is not sufficient to prove causality.

ChIP requires a high quantity of input samples, which is extremely hard to collect from carefully staged embryos. We have instead performed CUT&RUN which requires less starting material (Fig 6C-D, Fig EV3). The data is described in the result section line 340-347 and reads as follow:

"Looking at the peak scores associated with these gene regions, we detected increased Elba1 binding at the TSS region of cluster 2 genes, which were not visible at genes from the other clusters (Fig 6C). Intriguingly, this binding was reduced following heat shock (Fig 6C). CUT&RUN tracks over representative genomic loci are found in Fig EV3. We further aligned the consensus peaks and compared them to all upregulated genes. As before, the greatest overlap was detected between Elba1-GFP peaks and cluster 2 (Fig 6D)."

We are grateful for this comment as it has help us to substantially improve the manuscript.

3) In figure 2, the authors speculate that the increase of mir-309 cluster members in the very first stages is due to an early activity of Zelda. Zelda ChIPs are publicly available and the authors should check if the mir-309 cluster is bound by Zelda in the early embryo.

This has already been done by Fu et. al (PMID: 24764079). This work is referred to on row 159 in the manuscript.

4) How did the authors normalize for variation in the total amount of small RNAs? Have the authors assumed that total amounts were constant for each stage of embryogenesis or upon heat shock, and if yes how this assumption is justified?

The data is normalized to the library size (reads per million), and we have now made this clearer in the text, and made less conclusions regarding stage-to-stage differences. For example, (row 137-144)

"As expected during the maternal to zygotic transition, stage 3 is characterized by an increased proportion of degradation products ("other", Fig 2B) which then decrease at the end of MZT. We also noted that there is a larger proportion of rRNA fragments (rsRNAs) in stage 1 than in the other stages, while tRNA fragments (tsRNAs) are present at low levels at all stages (Fig 2B-C). In contrast, piRNAs are highly expressed throughout all stages. The most striking change, however, was the proportion of miRNA. It increased from approximately 9% in stage 1 embryos to over 50 % in stage 5 (Fig 2B). "

Our miRNA data do, however, align well with the data from Zhou et al. (Zhou et al, 2018). They looked at miRNA in *Drosophila* embryogenesis using a spike-in. Even though they don't have the same temporal resolution as us, their classification of maternal and zygotic miRNA largely agrees with ours. We have now added the following text in the manuscript to cover this question (row 233-239):

"To confirm that this classification was correct, we compared our results with the miRNA expression profiles of 0-2 and 2-4 h old embryos made by Zhou et al (Zhou et al, 2018), and found that our classifications largely aligns with theirs."

MINOR POINTS

Introduction:
- Drosophila embryogenesis lasts 22 hours, not 24 hours

The embryogenesis is temperature dependent. It takes 22-27 hours depending on the temperature. In 22 degrees we measured the embryogenesis to be approximately 27 hours. We have changed this in the introduction. On row 20 it now states 22-27 hours.

- The authors wrote: "it is easy to envision a general role for sncRNA, including miRNA, in initiating or influencing the early higher-order chromatin landscape". How miRNA could do that if not indirectly? It is possible that the authors hypothesized a change in rasiRNAs or in piRNAs and they could instead find a change in miRNAs upon heat shock. Nonetheless, a change in miRNA population cannot directly affect higher-order chromatin landscape as stated in the text. Therefore, the authors should remove "including miRNA".

We agree with the reviewer and have now deleted this part.

Figure 1:
- The authors wrote: "we found that the first two hours in embryogenesis are the most sensitive period for heat shock induction of long-term effects on heterochromatin". Maybe it would be more correct to claim that it is the only period? Indeed, heat shock performed by the authors in any other stage does not significantly affect eye pigmentation according to their results.

We agree with the reviewer and has now changed the text to (105-106):
…we found that the first two hours in embryogenesis is the only sensitive period for heat shock induction of long-term effects on heterochromatin.

- Figure 1B: 27h at 22C?

See answer above regarding the temperature-sensitivity of embryogenesis.

Figure 2:
- The authors wrote: "The first two hours of Drosophila embryogenesis, entailing stages 1-3 (at 22{degree sign} C)". Stage 3 lasts till 1h 20min AEL. From 1h 20min to 2h 10min AEL there is stage 4. The authors should change it with 1-4.

The text is correct considering that the embryos are kept at 22 degrees.

- Figure 2A: the authors should link embryonic stages with nuclear cycles inside the panel. Cycle 14 starts at 2h 10min AEL, therefore most of stage 4 (from cycle 10 to cycle 13) lays inside the first 2 hours of embryogenesis.

Thank you for pointing this out. We have now improved Fig2A with connecting lines to each mitotic cycle.

- Figure 2B and figure 2C could be merged, by simply add percentages to panel 2C for each RNA class. One extra panel could be added to show how many miRNAs, piRNAs tsRNAs, etc. are individually up

or downregulated in each embryonic stage as unique sequences, instead of showing the total amount that could be biased by few overrepresented members.

We agree that this could have been one option to visualize the results. However, as we in the current manuscript version describe the changes in sncRNA proportions, we believe that this is the most correct way of representing this data. Expression of unique sncRNA per stage can be found in Dataset EV1 (supplemental data 1 in earlier version).

Figure 3:
- From panel B to panel F the authors demonstrate the validity of their system and a lack of any temporal bias among groups. All this data can be moved to supplementary figures. Panel 3A could be included in Figure 4

We have done as suggested by the reviewer and moved this figure to the Expanded View. It is now Fig EV1. Old panel 3A is in the current manuscript partly integrated in Fig 3A (old figure 4A).

- Figure 3C: why the 2 y-axes are so much different in their RPKM ranges?

Because the genes that are not changed over nuclear cycle 14 are lowly expressed. We separated the graphs from each other to improve visibility.

Figure 4:
- Figure 4B is redundant with 4A. It could be removed

We agree that they are a bit redundant and has removed 4B. We keep 4A (now Fig 3B) since we think it is more informative.

- Figure 4C: together with miRNAs, all the other classes combined (all small RNAs minus miRNAs) should be shown in control and upon HS. Their lack of global difference would be a good comparison with the miRNAs, which are instead affected.

We are grateful for this comment and has included one such comparison together with the miRNAs (Fig 3C). The total sncRNA (minus miRNA) is non-significantly changed.

- Figure 4D: this panel is misleading as it shows only the RNAs that are significantly up or downregulated upon HS. It would be much better to show the whole populations as violin plots, together with the number of detected and significantly up or downregulated members per each RNA class. Indeed, the bimodal distributions observed in tsRNA and rsRNA simply represent the tails of of normally distributed samples. Furthermore, snoRNAs are mostly not affected as shown in supplementary figure 4. The 13 (modestly) upregulated snoRNAs out of 369 are clearly not representative of the whole population. Although specified in figure 4D, its representation is highly misleading for the reader. From supplementary figure 4, it is pretty clear that the only relevant effects of HS treatment are 1) global upregulation of several miRNA species; 2) up or downregulation of specific tsRNA species. These findings should be highlighted in the text and any other mention to snoRNAs or piRNAs should be removed, unless to claim for a lack of any significant effect on their populations upon HS treatment.

We agree with the reviewer and have now replaced this figure with a violin plot showing all unique sncRNA sequences (Fig 3D).

- Figure 4G: why only 3 zygotic miRNAs are shown in contrast to the several maternal miRNAs shown? Are those the only zygotic ones to be affected upon HS treatment?

Yes, that is correct. Of the miRNAs we classify as zygotically transcribed, we detect only 3 significantly upregulated.

- Supplementary 4H and 4I should be integrated in the main figure

These figures are now integrated in Fig 3F and G.

- If transcription of zygotic miRNA is affected by heat shock treatment, how do the authors explain that zelda-dependent mir-309 cluster is not affected?

We mainly find maternally loaded miRNA to be upregulated by heat shock. Only three zygotic miRNA is increased. We don't have a good explanation why only these three are upregulated, but since the miR-309 cluster is not, it is tempting to speculate that these zygotic miRNAs are co-regulated by other factors such as Dorsal.

Figure 5:

- Figure 5B: Among all the 459 downregulated genes upon HS treatment, the authors should check for any overlap with a list of all the known suppression of variegation genes, as they did for the few genes shown in 5B. Similarly, the authors should compare the list of the 1134 upregulated genes with enhancer of variegation genes. Finding that other genes involved in the variegation are also affected by the heat shock could help to explain the additional contributions not linked to Elba1 function.

We have now included a comparison of up- and downregulated genes to genes included in the GO-term GO:0031507 (heterochromatin formation) (Fig4C). We found no enrichment in either group, and elba1 and pgc (both downregulated) were the only genes from this GO-term reaching statistical significance and a Log2 fold change < -1.

- Figure 5J: the authors should show a representative picture of Elba1-GFP embryos upon heat shock treatment

We agree with the reviewer and have now also included a representative image of a heat shocked embryo alongside a control embryo (Fig 5A).

Thank you for sending us your revised manuscript. We have now heard back from the two reviewers who were asked to evaluate your revised study. As you will see below, the reviewers think that the performed revisions have strengthened the study. While reviewer #2 is supportive of publication, reviewer #1 still lists some remaining concerns, which we would ask you to address in an exceptional final round of revision.

Specifically, reviewer #1 refers to some additional evidence that needs to be provided to better support that Elba1 is directly targeted by miRNAs. Moreover, they mention a missing control in the Ago-IP qPCR experiment and refer to the need to present further quantifications related to the CUT&RUN analyses. The remaining issues raised by reviewer #1 and the minor issues raised by reviewer #2 would all need to be convincingly addressed.

Reviewer #1:

The revised manuscript is much improved, in terms of flow and readability. I appreciate that the authors put a lot of work into performing new experiments to support their claims. Unfortunately there are some methodological issues, which call the conclusions into question. Without these pieces of data, as before, I think the authors would need to moderate their overall conclusions.

Fig 5C - I suggest the authors refer to Bartel 2018 regarding the nuances of miRNA targeting
https://www.sciencedirect.com/science/article/pii/S0092867418302861

As it stands, the analysis was not appropriately done. The seed sequences that one downloads from TargetScan are *7-base* sequences of the miRNA, meaning that the target region on the mRNA should be the reverse complement (an 8-mer match would refer to a 7-bp complementary region in the mRNA followed by an "A" base that is not necessarily complementary to the miRNA). So both of the putative miRNA target sites that are highlighted in red in the 3'UTR of elba1 are in the incorrect orientation, i.e. not real target sites.

1-base mismatches to the seed sequence are generally not tolerated, unless they occur at position 8 relative to the miRNA seed, or are accompanied by 3' compensatory base-pairing outside of the seed, so I presume that would be the minority of the annotated antisense positions in the figure.

This leaves just 2 candidate miRNA target sites in the CDS (miR-2a-2-5p and miR-13b-1-5p), and CDS targeting is typically marginally if at all effective. I would call this extremely weak evidence supporting elba1 targeting by miRNAs

Fig 5F - It seems that no reference gene was used with the Ago-IP qPCR, so it is difficult to interpret the enrichment of elba1 in the Ago-IP sample, since RNA binding proteins will tend to stick to mRNA non-specifically

Fig 6C - the CUT&RUN patterns in control vs heat shock are qualitatively similar, so it is difficult to discern that cluster 2 binding changes more than the other clusters; and the browser tracks in the supplement are quite similar looking. Can this difference be more rigorously quantified? The text mentions comparison of "peak scores," but these do not seem to be compared anywhere in the figures. The methods mention the use of a spike-in, but it's not clear how that spike-in factored into how the binding intensities in control vs heat shock were compared.

Reviewer #2:

In this revised manuscript, Örkenby and colleagues carefully answered to our expressed concerns.
Before publication, the authors should carefully check their reference list and correctly place the right staged embryos in one of the figures.

Details:

-In line 120 the authors shall include the reference to this work:
Polycomb-Dependent Chromatin Looping Contributes to Gene Silencing during Drosophila Development
Yuki Ogiyama 1 , Bernd Schuettengruber 2 , Giorgio L Papadopoulos 1 , Jia-Ming Chang 3 , Giacomo Cavalli

-In line 122 the authors shall include the reference to this work:
HP1 drives de novo 3D genome reorganization in early Drosophila embryos
Fides Zenk, Yinxiu Zhan, Pavel Kos, Eva Löser, Nazerke Atinbayeva, Melanie Schächtle, Guido Tiana, Luca Giorgetti & Nicola Iovino

-Figure 5a Heat shock embryo. This embryo is not at stage 5; the image shall be replaced with a more correctly staged stage 5 embryo.

Reviewer #1:

The revised manuscript is much improved, in terms of flow and readability. I appreciate that the authors put a lot of work into performing new experiments to support their claims. Unfortunately there are some methodological issues, which call the conclusions into question. Without these pieces of data, as before, I think the authors would need to moderate their overall conclusions.

We are pleased that you found the manuscript to be improved and appreciate your thorough review that helped enhance it. In this version, we addressed your remaining methodological concerns and moderated our conclusions about miRNA targeting of Elba1. Please see the point-to-point answers below for details.

Fig 5C - I suggest the authors refer to Bartel 2018 regarding the nuances of miRNA targeting https://www.sciencedirect.com/science/article/pii/S0092867418302861

As it stands, the analysis was not appropriately done. The seed sequences that one downloads from TargetScan are *7-base* sequences of the miRNA, meaning that the target region on the mRNA should be the reverse complement (an 8-mer match would refer to a 7-bp complementary region in the mRNA followed by an "A" base that is not necessarily complementary to the miRNA). So both of the putative miRNA target sites that are highlighted in red in the 3'UTR of elba1 are in the incorrect orientation, i.e. not real target sites.

1-base mismatches to the seed sequence are generally not tolerated, unless they occur at position 8 relative to the miRNA seed, or are accompanied by 3' compensatory base-pairing outside of the seed, so I presume that would be the minority of the annotated antisense positions in the figure.

This leaves just 2 candidate miRNA target sites in the CDS (miR-2a-2-5p and miR-13b-1-5p), and CDS targeting is typically marginally if at all effective. I would call this extremely weak evidence supporting elba1 targeting by miRNAs

We appreciate the feedback and have redone the seed target analysis using TargetScan Fly's script, based on Agarwal et. al's research on miRNA targeting in *Drosophila* (Agarwal *et al* (2018) PMID: 30286781). The detected target site types (7mer-m8, 7mer-A1, and 6mer) were color-coded as shown in Fig 5C. Importantly, we detected one miRNA (miR-283-3p), to have a candidate targeting 6mer site to the 3'UTR. In addition to this miRNA, we found multiple miRNAs with candidate targeting sites in the CDS. Similar to mammals, miRNA targeting to the 3'UTR is most effective, but seed-binding sites within the CDS also have some effect (Agarwal *et. al* (2018) PMID: 30286781).

In general, the fly 3′ UTRs are shorter and have a higher AU content than those of mammals. In this context it is worth noting that Elba1 has a short ORF (1092 bp) and 3'UTR (172 bp), likely increasing miRNA target efficiency on this transcript (Agarwal *et al* 2018 PMID: 30286781).

We have updated the text accordingly in the manuscript (rows 265-274).

Fig 5F - It seems that no reference gene was used with the Ago-IP qPCR, so it is difficult to interpret the enrichment of elba1 in the Ago-IP sample, since RNA binding proteins will tend to stick to mRNA non-specifically

We have now performed qPCR with a reference gene (RpL32), both on the samples reported in the last version of the manuscript, as well as on a new set of samples. The graph in the new version of the manuscript therefore contains more data points as well as data for the reference gene. We are pleased to report that our conclusion remains unchanged. However, as the reviewer noted, we can confirm that Ago1 is somewhat "sticky", but the degree of enrichment is not comparable to what is observed with Elba1 (see Fig 5F).

Based on the miRNA seed binding regions on the Elba1 transcript, the loss of Elba1 reduction following heat shock in Dcr-1 knockdown flies, and strong negative correlation between miRNAs and Elba1, our data strongly support the hypothesis that Elba1 is targeted by miRNA under heat shock stress. However, we cannot exclude that some of the effect is indirect and therefore recognize that some of our statements may have been overly assertive and have adjusted them accordingly.

To modify our statements, we have made the following changes:

1. **The title is changed to "Stress-sensitive dynamics of miRNAs and Elba1 in *Drosophila* embryogenesis".**
2. **We have added "direct or indirect" to the last sentence in the abstract. It now reads "We propose that maternal miRNAs, retained in response to an early embryonic heat shock, shape the subsequent de novo heterochromatin establishment that occurs during early development via direct or indirect regulation of some of the earliest expressed genes, including Elba1."**
3. **This part "thus indicative of a stress-sensitive miRNA-Elba1-axis" is deleted from the discussion (row 357).**

Fig 6C - the CUT&RUN patterns in control vs heat shock are qualitatively similar, so it is difficult to discern that cluster 2 binding changes more than the other clusters; and the browser tracks in the supplement are quite similar looking. Can this difference be more rigorously quantified? The text mentions comparison of "peak scores," but these do not seem to be compared anywhere in the figures. The methods mention the use of a spike-in, but it's not clear how that spike-in factored into how the binding intensities in control vs heat shock were compared.

We agree with the reviewer that the track profiles, but not the intensities, of the two conditions are similar. In the revised manuscript, we have therefore clarified this in the text (rows 314-318). We have also included two new sub-figures to fig EV3 (EV3A and EV3B), which shows the score profiles over TSS per cluster, and the mean peak scores 250bp upstream TSS up to TSS per cluster, respectively. These figures highlight that Elba1 has the highest enrichment at TSS in cluster 2 genes compared to the other clusters, which is further emphasized in the newly replaced IGV track figures (former EV3A and B, now EV3C and D). Our original conclusion remains the same - genes in cluster 2 do have a more pronounced Elba1 binding at the TSS region compared to other clusters, and this binding decreases after heat shock. We hope that the new figures will make these findings clearer.

To normalize the data, the spike-in was added in equal concentrations (via the stop buffer) to all samples. The spike-in data was thereafter used to calculate scaling factors within the nf-core CUT&RUN pipeline. This has been further specified in the material and methods (rows 729, 737). The downstream analysis was conducted on this normalized data. The primary antibody (GFP) used in Elba1-GFP embryos was also used in *w1118* flies as a negative control, as they should not express GFP.

Reviewer #2:

In this revised manuscript, Örkenby and colleagues carefully answered to our expressed concerns.
Before publication, the authors should carefully check their reference list and correctly place the right staged embryos in one of the figures.

We are happy to hear that we have answered your previous concerns. We believe that we have properly addressed the new comments and concerns as well. See details below.

Details:

-In line 120 the authors shall include the reference to this work:
Polycomb-Dependent Chromatin Looping Contributes to Gene Silencing during Drosophila Development
Yuki Ogiyama 1 , Bernd Schuettengruber 2 , Giorgio L Papadopoulos 1 , Jia-Ming Chang 3 , Giacomo Cavalli

-In line 122 the authors shall include the reference to this work:
HP1 drives de novo 3D genome reorganization in early Drosophila embryos
Fides Zenk, Yinxiu Zhan, Pavel Kos, Eva Löser, Nazerke Atinbayeva, Melanie Schächtle, Guido Tiana, Luca Giorgetti & Nicola Iovino

Thank you for these suggestions, they are now included as requested in the new version of the manuscript.

-Figure 5a Heat shock embryo. This embryo is not at stage 5; the image shall be replaced with a more correctly staged stage 5 embryo.

The heat-shocked embryo in Fig 5A has been replaced with a carefully staged stage 5 embryo from the same batch as the control.

2nd Revision - Editorial Decision 23rd Feb 2023

Thank you again for sending us your revised manuscript. We think that the inclusion of the additional analyses and clarifications, as well as toning down statements regarding the targeting of Elba1 by miRNAs have satisfactorily addressed the remaining concerns of the reviewers. Overall, we are now satisfied with the modifications made and I am pleased to inform you that your study has been accepted for publication.

EMBO Press Author Checklist

Corresponding Author Name: Anita Öst	
Journal Submitted to: Molecular Systems Biology	
Manuscript Number: MSB-2022-11148R	

USEFUL LINKS FOR COMPLETING THIS FORM
The EMBO Journal - Author Guidelines
EMBO Reports - Author Guidelines
Molecular Systems Biology - Author Guidelines
EMBO Molecular Medicine - Author Guidelines

Reporting Checklist for Life Science Articles (updated January
This checklist is adapted from Materials Design Analysis Reporting (MDAR) Checklist for Authors. MDAR establishes a minimum set of requirements in transparent reporting in the life sciences (see Statement of Task: 10.31222/osf.io/9sm4x). Please follow the journal's guidelines in preparing your
Please note that a copy of this checklist will be published alongside your article.

Abridged guidelines for figures
1. Data
The data shown in figures should satisfy the following conditions:
- the data were obtained and processed according to the field's best practice and are presented to reflect the results of the experiments in an accurate and unbiased manner.
- ideally, figure panels should include only measurements that are directly comparable to each other and obtained with the same assay.
- plots include clearly labeled error bars for independent experiments and sample sizes. Unless justified, error bars should not be shown for technical
- if n<5, the individual data points from each experiment should be plotted. Any statistical test employed should be justified.
- Source Data should be included to report the data underlying figures according to the guidelines set out in the authorship guidelines on Data

2. Captions
Each figure caption should contain the following information, for each panel where they are relevant:
- a specification of the experimental system investigated (eg cell line, species name).
- the assay(s) and method(s) used to carry out the reported observations and measurements.
- an explicit mention of the biological and chemical entity(ies) that are being measured.
- an explicit mention of the biological and chemical entity(ies) that are altered/varied/perturbed in a controlled manner.
- the exact sample size (n) for each experimental group/condition, given as a number, not a range;
- a description of the sample collection allowing the reader to understand whether the samples represent technical or biological replicates (including how many animals, litters, cultures, etc.).
- a statement of how many times the experiment shown was independently replicated in the laboratory.
- definitions of statistical methods and measures:
  - common tests, such as t-test (please specify whether paired vs. unpaired), simple χ2 tests, Wilcoxon and Mann-Whitney tests, can be unambiguously identified by name only, but more complex techniques should be described in the methods section;
  - are tests one-sided or two-sided?
  - are there adjustments for multiple comparisons?
  - exact statistical test results, e.g., P values = x but not P values < x;
  - definition of 'center values' as median or average;
  - definition of error bars as s.d. or s.e.m.

Please complete ALL of the questions below.
Select "Not Applicable" only when the requested information is not relevant for your study.

Materials

Newly Created Materials	Information included in the manuscript?	In which section is the information available? (Reagents and Tools Table, Materials and Methods, Figures, Data Availability Section)
New materials and reagents need to be available; do any restrictions apply?	Not Applicable	

Antibodies	Information included in the manuscript?	In which section is the information available? (Reagents and Tools Table, Materials and Methods, Figures, Data Availability Section)
For **antibodies** provide the following information:<br>- Commercial antibodies: RRID (if possible) or supplier name, catalogue number and or/clone number<br>- Non-commercial: RRID or citation	Yes	Material and methods

DNA and RNA sequences	Information included in the manuscript?	In which section is the information available? (Reagents and Tools Table, Materials and Methods, Figures, Data Availability Section)
**Short novel DNA or RNA including primers, probes**: provide the sequences.	Yes	Material and methods

Cell materials	Information included in the manuscript?	In which section is the information available? (Reagents and Tools Table, Materials and Methods, Figures, Data Availability Section)
**Cell lines:** Provide species information, strain. Provide accession number in repository **OR** supplier name, catalog number, clone number, and/**OR** RRID.	Not Applicable	
**Primary cultures:** Provide species, strain, sex of origin, genetic modification status.	Not Applicable	
Report if the cell lines were recently **authenticated** (e.g., by STR profiling) and tested for mycoplasma contamination.	Not Applicable	

Experimental animals	Information included in the manuscript?	In which section is the information available? (Reagents and Tools Table, Materials and Methods, Figures, Data Availability Section)
**Laboratory animals or Model organisms:** Provide species, strain, sex, age, genetic modification status. Provide accession number in repository **OR** supplier name, catalog number, clone number, **OR** RRID.	Yes	Material and methods, Figures
**Animal observed in or captured from the field:** Provide species, sex, and age where possible.	Not Applicable	
Please detail **housing and husbandry conditions**.	Yes	Material and methods

Plants and microbes	Information included in the manuscript?	In which section is the information available? (Reagents and Tools Table, Materials and Methods, Figures, Data Availability Section)
**Plants:** provide species and strain, ecotype and cultivar where relevant, unique accession number if available, and source (including location for collected wild specimens).	Not Applicable	
**Microbes:** provide species and strain, unique accession number if available, and source.	Not Applicable	

Human research participants	Information included in the manuscript?	In which section is the information available? (Reagents and Tools Table, Materials and Methods, Figures, Data Availability Section)
If collected and within the bounds of privacy constraints report on age, sex and gender or ethnicity for all study participants.	Not Applicable	

Core facilities	Information included in the manuscript?	In which section is the information available? (Reagents and Tools Table, Materials and Methods, Figures, Data Availability Section)
If your work benefited from core facilities, was their service mentioned in the acknowledgments section?	Yes	Acknowledgments

Design

Study protocol	Information included in the manuscript?	In which section is the information available? (Reagents and Tools Table, Materials and Methods, Figures, Data Availability Section)
If study protocol has been **pre-registered, provide DOI in the manuscript**. For clinical trials, provide the trial registration number **OR** cite DOI.	Not Applicable	
Report the **clinical trial registration number** (at ClinicalTrials.gov or equivalent), where applicable.	Not Applicable	

Laboratory protocol	Information included in the manuscript?	In which section is the information available? (Reagents and Tools Table, Materials and Methods, Figures, Data Availability Section)
Provide DOI OR other citation details if **external detailed step-by-step protocols** are available.	Yes	Material and methods

Experimental study design and statistics	Information included in the manuscript?	In which section is the information available? (Reagents and Tools Table, Materials and Methods, Figures, Data Availability Section)
Include a statement about **sample size** estimate even if no statistical methods were used.	Not Applicable	
Were any steps taken to minimize the effects of subjective bias when allocating animals/samples to treatment (e.g. **randomization procedure**)? If yes, have they been described?	Yes	Material and methods
Include a statement about **blinding** even if no blinding was done.	Not Applicable	
Describe **inclusion/exclusion criteria** if samples or animals were excluded from the analysis. Were the criteria pre-established?<br><br>If sample or data points were omitted from analysis, report if this was due to attrition or intentional exclusion and provide justification.	Yes	Material and methods
For every figure, are **statistical tests** justified as appropriate? Do the data meet the assumptions of the tests (e.g., normal distribution)? Describe any methods used to assess it. Is there an estimate of variation within each group of data? Is the variance similar between the groups that are being statistically compared?	Yes	Material and methods, figures

Sample definition and in-laboratory replication	Information included in the manuscript?	In which section is the information available? (Reagents and Tools Table, Materials and Methods, Figures, Data Availability Section)
In the figure legends: state number of times the experiment was **replicated** in laboratory.	Yes	Figure legends, Material and methods
In the figure legends: define whether data describe **technical or biological replicates**.	Yes	Figure legends

Ethics

Ethics	Information included in the manuscript?	In which section is the information available? (Reagents and Tools Table, Materials and Methods, Figures, Data Availability Section)
Studies involving **human participants**: State details of **authority granting ethics approval** (IRB or equivalent committee(s), provide reference number for approval.	Not Applicable	
Studies involving **human participants**: Include a statement confirming that **informed consent** was obtained from all subjects and that the experiments conformed to the principles set out in the WMA Declaration of Helsinki and the Department of Health and Human Services Belmont Report.	Not Applicable	
Studies involving **human participants:** For publication of **patient photos**, include a statement confirming that consent to publish was obtained.	Not Applicable	
Studies involving experimental **animals**: State details of **authority granting ethics approva**l (IRB or equivalent committee(s), provide reference number for approval. Include a statement of compliance with ethical regulations.	Not Applicable	
Studies involving **specimen and field samples:** State if relevant **permits** obtained, provide details of authority approving study; if none were required, explain why.	Not Applicable	

Dual Use Research of Concern (DURC)	Information included in the manuscript?	In which section is the information available? (Reagents and Tools Table, Materials and Methods, Figures, Data Availability Section)
Could your study fall under dual use research restrictions? Please check biosecurity documents and list of **select agents and toxins** (CDC): https://www.selectagents.gov/sat/list.htm	Not Applicable	
If you used a select agent, is the security level of the lab appropriate and reported in the manuscript?	Not Applicable	
If a study is subject to dual use research of concern regulations, is the name of the **authority granting approval and reference number** for the regulatory approval provided in the manuscript?	Not Applicable	

Reporting

The MDAR framework recommends adoption of discipline-specific guidelines, established and endorsed through community initiatives. Journals have their own policy about requiring specific guidelines and recommendations to complement MDAR.

Adherence to community standards	Information included in the manuscript?	In which section is the information available? (Reagents and Tools Table, Materials and Methods, Figures, Data Availability Section)
State if relevant guidelines or checklists (e.g., **ICMJE, MIBBI, ARRIVE, PRISMA**) have been followed or provided.	Not Applicable	
For **tumor marker prognostic studies,** we recommend that you follow the **REMARK** reporting guidelines (see link list at top right). See author guidelines, under 'Reporting Guidelines'. Please confirm you have followed these guidelines.	Not Applicable	
For **phase II and III randomized controlled trials**, please refer to the **CONSORT** flow diagram (see link list at top right) and submit the CONSORT checklist (see link list at top right) with your submission. See author guidelines, under 'Reporting Guidelines'. Please confirm you have submitted this list.	Not Applicable	

Data Availability

Data availability	Information included in the manuscript?	In which section is the information available? (Reagents and Tools Table, Materials and Methods, Figures, Data Availability Section)
Have **primary datasets** been deposited according to the journal's guidelines (see 'Data Deposition' section) and the respective accession numbers provided in the Data Availability Section?	Yes	Data availability
Were **human clinical and genomic datasets** deposited in a public access-controlled repository in accordance to ethical obligations to the patients and to the applicable consent agreement?	Not Applicable	
Are **computational models** that are central and integral to a study available without restrictions in a machine-readable form? Were the relevant accession numbers or links provided?	Not Applicable	
If publicly available data were reused, provide the respective **data citations in the reference list**.	Yes	Results, Discussion, Material and Methods, Figure legends, References
